# A programmable synthetic lineage-control network that differentiates human IPSCs into glucose-sensitive insulin-secreting beta-like cells

**DOI:** 10.1038/ncomms11247

**Published:** 2016-04-11

**Authors:** Pratik Saxena, Boon Chin Heng, Peng Bai, Marc Folcher, Henryk Zulewski, Martin Fussenegger

**Affiliations:** 1 Department of Biosystems Science and Engineering, ETH Zurich, Mattenstrasse 26, CH-4058 Basel, Switzerland; 2 Division of Endocrinology, Diabetes and Metabolism, University Hospital Basel, Petersgraben 4, CH-4031 Basel, Switzerland; 3 Faculty of Science, University of Basel, Mattenstrasse 26, CH-4058 Basel, Switzerland

## Abstract

Synthetic biology has advanced the design of standardized transcription control devices that programme cellular behaviour. By coupling synthetic signalling cascade- and transcription factor-based gene switches with reverse and differential sensitivity to the licensed food additive vanillic acid, we designed a synthetic lineage-control network combining vanillic acid-triggered mutually exclusive expression switches for the transcription factors Ngn3 (neurogenin 3; OFF-ON-OFF) and Pdx1 (pancreatic and duodenal homeobox 1; ON-OFF-ON) with the concomitant induction of MafA (V-maf musculoaponeurotic fibrosarcoma oncogene homologue A; OFF-ON). This designer network consisting of different network topologies orchestrating the timely control of transgenic and genomic Ngn3, Pdx1 and MafA variants is able to programme human induced pluripotent stem cells (hIPSCs)-derived pancreatic progenitor cells into glucose-sensitive insulin-secreting beta-like cells, whose glucose-stimulated insulin-release dynamics are comparable to human pancreatic islets. Synthetic lineage-control networks may provide the missing link to genetically programme somatic cells into autologous cell phenotypes for regenerative medicine.

## Abstract (web summary)

Synthetic biology offers the potential for the design and implementation of rationally designed, complex genetic programmes. Here the authors design a genetic network to trigger the differentiation of patient derived IPSCs into beta-like cells.

## Graphical abstract

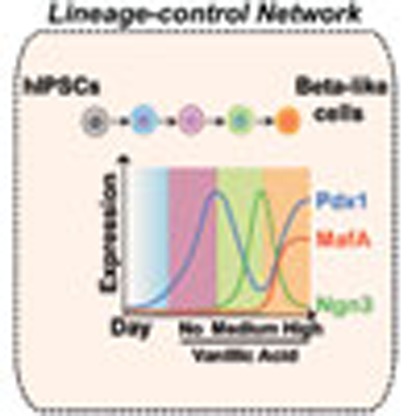

Cell-fate decisions during development are regulated by various mechanisms, including morphogen gradients, regulated activation and silencing of key transcription factors, microRNAs, epigenetic modification and lateral inhibition. The latter implies that the decision of one cell to adopt a specific phenotype is associated with the inhibition of neighbouring cells to enter the same developmental path. In mammals, insights into the role of key transcription factors that control development of highly specialized organs like the pancreas were derived from experiments in mice, especially various genetically modified animals^1^,^2^,^3^,^4^. Normal development of the pancreas requires the activation of pancreatic duodenal homeobox protein (Pdx1) in pre-patterned cells of the endoderm. Inactivating mutations of *Pdx1* are associated with pancreas agenesis in mouse and humans^5^,^6^. A similar cell fate decision occurs later with the activation of Ngn3 that is required for the development of all endocrine cells in the pancreas^7^. Absence of Ngn3 is associated with the loss of pancreatic endocrine cells, whereas the activation of Ngn3 not only allows the differentiation of endocrine cells but also induces lateral inhibition of neighbouring cells—via Delta-Notch pathway—to enter the same pancreatic endocrine cell fate^8^. This Ngn3-mediated cell-switch occurs at a specific time point and for a short period of time in mice^9^. Thereafter, it is silenced and becomes almost undetectable in postnatal pancreatic islets. Conversely, Pdx1-positive Ngn3-positive cells reduce Pdx1 expression, as Ngn3-positive cells are Pdx1 negative^10^. They re-express Pdx1, however, as they go on their path towards glucose-sensitive insulin-secreting cells with parallel induction of MafA that is required for proper differentiation and maturation of pancreatic beta cells^11^. Data supporting these expression dynamics are derived from mice experiments^1^,^11^,^12^. A synthetic gene-switch governing cell fate decision in human induced pluripotent stem cells (hIPSCs) could facilitate the differentiation of glucose-sensitive insulin-secreting cells.

In recent years, synthetic biology has significantly advanced the rational design of synthetic gene networks that can interface with host metabolism, correct physiological disturbances^13^ and provide treatment strategies for a variety of metabolic disorders, including gouty arthritis^14^, obesity^15^ and type-2 diabetes^16^. Currently, synthetic biology principles may provide the componentry and gene network topologies for the assembly of synthetic lineage-control networks that can programme cell-fate decisions and provide targeted differentiation of stem cells into terminally differentiated somatic cells. Synthetic lineage-control networks may therefore provide the missing link between human pluripotent stem cells^17^ and their true impact on regenerative medicine^18^,^19^,^20^. The use of autologous stem cells in regenerative medicine holds great promise for curing many diseases, including type-1 diabetes mellitus (T1DM), which is characterized by the autoimmune destruction of insulin-producing pancreatic beta cells, thus making patients dependent on exogenous insulin to control their blood glucose^21^,^22^. Although insulin therapy has changed the prospects and survival of T1DM patients, these patients still suffer from diabetic complications arising from the lack of physiological insulin secretion and excessive glucose levels^23^. The replacement of the pancreatic beta cells either by pancreas transplantation or by transplantation of pancreatic islets has been shown to normalize blood glucose and even improve existing complications of diabetes^24^. However, insulin independence 5 years after islet transplantation can only be achieved in up to 55% of the patients even when using the latest generation of immune suppression strategies^25^,^26^. Transplantation of human islets or the entire pancreas has allowed T1DM patients to become somewhat insulin independent, which provides a proof-of-concept for beta-cell replacement therapies^27^,^28^. However, because of the shortage of donor pancreases and islets, as well as the significant risk associated with transplantation and life-long immunosuppression, the rational differentiation of stem cells into functional beta-cells remains an attractive alternative^29^,^30^. Nevertheless, a definitive cure for T1DM should address both the beta-cell deficit and the autoimmune response to cells that express insulin. Any beta-cell mimetic should be able to store large amounts of insulin and secrete it on demand, as in response to glucose stimulation^29^,^31^. The most effective protocols for the *in vitro* generation of *bonafide* insulin-secreting beta-like cells that are suitable for transplantation have been the result of sophisticated trial-and-error studies elaborating timely addition of complex growth factor and small-molecule compound cocktails to human pancreatic progenitor cells^32^,^33^,^34^. The differentiation of pancreatic progenitor cells to beta-like cells is the most challenging part as current protocols provide inconsistent results and limited success in programming pancreatic progenitor cells into glucose-sensitive insulin-secreting beta-like cells^35^,^36^,^37^. One of the reasons for these observations could be the heterogeneity in endocrine differentiation and maturation towards a beta cell phenotype. Here we show that a synthetic lineage-control network programming the dynamic expression of the transcription factors Ngn3, Pdx1 and MafA enables the differentiation of hIPSC-derived pancreatic progenitor cells to glucose-sensitive insulin-secreting beta-like cells (Supplementary Fig. 1).

## Results

### Vanillic acid-programmable positive band-pass filter

The differentiation pathway from pancreatic progenitor cells to glucose-sensitive insulin-secreting pancreatic beta-cells combines the transient mutually exclusive expression switches of Ngn3 (OFF-ON-OFF) and Pdx1 (ON-OFF-ON) with the concomitant induction of MafA (OFF-ON) expression^10^,^11^. Since independent control of the pancreatic transcription factors Ngn3, Pdx1 and MafA by different antibiotic transgene control systems responsive to tetracycline, erythromycin and pristinamycin did not result in the desired differential control dynamics (Supplementary Fig. 2), we have designed a vanillic acid-programmable synthetic lineage-control network that programmes hIPSC-derived pancreatic progenitor cells to specifically differentiate into glucose-sensitive insulin-secreting beta-like cells in a seamless and self-sufficient manner. The timely coordination of mutually exclusive Ngn3 and Pdx1 expression with MafA induction requires the trigger-controlled execution of a complex genetic programme that orchestrates two overlapping antagonistic band-pass filter expression profiles (OFF-ON-OFF and ON-OFF-ON), a positive band-pass filter for Ngn3 (OFF-ON-OFF) and a negative band-pass filter, also known as band-stop filter, for Pdx1 (ON-OFF-ON), the ramp-up expression phase of which is linked to a graded induction of MafA (OFF-ON).

**Figure 1:**
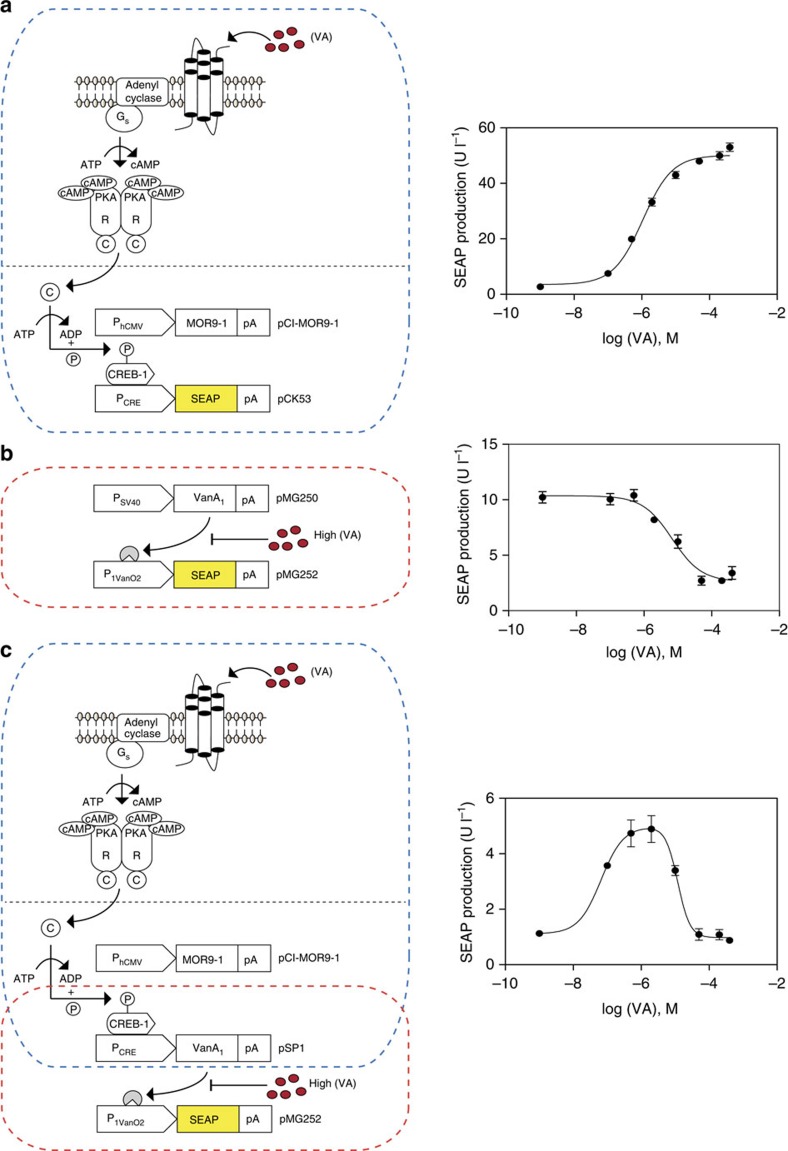
Design of a vanillic acid-responsive positive band-pass filter providing an OFF-ON-OFF expression profile. (**a**) Vanillic acid-inducible transgene expression. The constitutively expressed vanillic acid-sensitive olfactory G protein-coupled receptor MOR9-1 (pCI-MOR9-1; P_hCMV_-MOR9-1-pA) senses extracellular vanillic acid levels and triggers G protein (G_s_)-mediated activation of the membrane-bound adenylyl cyclase (AC) that converts ATP into cyclic AMP (cAMP). The resulting intracellular cAMP surge activates PKA (protein kinase A), whose catalytic subunits translocate into the nucleus to phosphorylate cAMP response element-binding protein 1 (CREB1). Activated CREB1 binds to synthetic promoters (P_CRE_) containing cAMP-response elements (CRE) and induces P_CRE_-driven expression of human placental secreted alkaline phosphatase (SEAP; pCK53, P_CRE_-SEAP-pA). Co-transfection of pCI-MOR9-1 and pCK53 into human mesenchymal stem cells (hMSC-TERT) grown for 48 h in the presence of increasing vanillic acid concentrations results in a dose-inducible SEAP expression profile. (**b**) Vanillic acid-repressible transgene expression. The constitutively expressed, vanillic acid-dependent transactivator VanA_1_ (pMG250, P_SV40_-VanA_1_-pA, VanA_1_, VanR-VP16) binds and activates the chimeric promoter P_1VanO2_ (pMG252, P_1VanO2_-SEAP-pA) in the absence of vanillic acid. In the presence of increasing vanillic acid concentrations, VanA_1_ is released from P_1VanO2_, and transgene expression is shut down. Co-transfection of pMG250 and pMG252 into hMSC-TERT grown for 48 h in the presence of increasing vanillic acid concentrations results in a dose-repressible SEAP expression profile. (**c**) Positive band-pass expression filter. Serial interconnection of the synthetic vanillic acid-inducible signalling cascade (**a**) with the vanillic acid-repressible transcription factor-based gene switch (**b**) by P_CRE_-mediated expression of VanA_1_ (pSP1, P_CRE_-VanA_1_-pA) results in a two-level feed-forward cascade. Owing to the opposing responsiveness and differential sensitivity to vanillic acid, this synthetic gene network programmes SEAP expression with a positive band-pass filter profile (OFF-ON-OFF) as vanillic acid levels are increased. Medium vanillic acid levels activate MOR9-1, which induces P_CRE_-driven VanA_1_ expression. VanA_1_ remains active and triggers P_1VanO2_-mediated SEAP expression in feed-forward manner, which increases to maximum levels. At high vanillic acid concentrations, MOR9-1 maintains P_CRE_-driven VanA_1_ expression, but the transactivator dissociates from P_1VanO2_, which shuts SEAP expression down. Co-transfection of pCI-MOR9-1, pSP1 and pMG252 into hMSC-TERT grown for 48 h in the presence of increasing vanillic acid concentrations programmes SEAP expression with a positive band-pass profile (OFF-ON-OFF). Data are the means±s.d. of triplicate experiments (*n*=9).

The core of the synthetic lineage-control network consists of two transgene control devices that are sensitive to the food component and licensed food additive vanillic acid. These devices are a synthetic vanillic acid-inducible (ON-type) signalling cascade that is gradually induced by increasing the vanillic acid concentration and a vanillic acid-repressible (OFF-type) gene switch that is repressed in a vanillic acid dose-dependent manner (Fig. 1a,b). The designer cascade consists of the vanillic acid-sensitive mammalian olfactory receptor MOR9-1, which sequentially activates the G protein Sα (G_Sα_) and adenylyl cyclase to produce a cyclic AMP (cAMP) second messenger surge^38^ that is rewired via the cAMP-responsive protein kinase A-mediated phospho-activation of the cAMP-response element-binding protein 1 (CREB1) to the induction of synthetic promoters (P_CRE_) containing CREB1-specific cAMP response elements (CRE; Fig. 1a). The co-transfection of **pCI-MOR9-1** (P_hCMV_-MOR9-1-pA_SV40_) and **pCK53** (P_CRE_-SEAP-pA_SV40_) into human mesenchymal stem cells (hMSC-TERT) confirmed the vanillic acid-adjustable secreted alkaline phosphatase (SEAP) induction of the designer cascade (>10 nM vanillic acid; Fig. 1a). The vanillic acid-repressible gene switch consists of the vanillic acid-dependent transactivator (VanA_1_), which binds and activates vanillic acid-responsive promoters (for example, P_1VanO2_) at low and medium vanillic acid levels (<2 μM). At high vanillic acid concentrations (>2 μM), VanA_1_ dissociates from P_1VanO2_, which results in the dose-dependent repression of transgene expression^39^ (Fig. 1b). The co-transfection of **pMG250** (P_SV40_-VanA_1_-pA_SV40_) and **pMG252** (P_1VanO2_-SEAP-pA_SV40_) into hMSC-TERT corroborated the fine-tuning of the vanillic acid-repressible SEAP expression (Fig. 1b).

The opposing responsiveness and differential sensitivity of the control devices to vanillic acid are essential to programme band-pass filter expression profiles. Upon daisy-chaining the designer cascade (**pCI-MOR9-1**; P_hCMV_-MOR9-1-pA_SV40_; **pSP1**, P_CRE_-VanA_1_-pA_SV40_) and the gene switch (**pSP1**, P_CRE_-VanA_1_-pA_SV40_; **pMG252**, P_1VanO2_-SEAP-pA_SV40_) in the same cell, the network executes a band-pass filter SEAP expression profile when exposed to increasing concentrations of vanillic acid (Fig. 1c). Medium vanillic acid levels (10 nM to 2 μM) activate MOR9-1, which induces P_CRE_-driven VanA_1_ expression. VanA_1_ remains active within this concentration range and, in a feed-forward amplifier manner, triggers P_1VanO2_-mediated SEAP expression, which gradually increases to maximum levels (Fig. 1c). At high vanillic acid concentrations (2 μM to 400 μM), MOR9-1 maintains P_CRE_-driven VanA_1_ expression, but the transactivator is inactivated and dissociates from P_1VanO2_, which results in the gradual shutdown of SEAP expression (Fig. 1c).

### Vanillic acid-programmable lineage-control network

**Figure 2:**
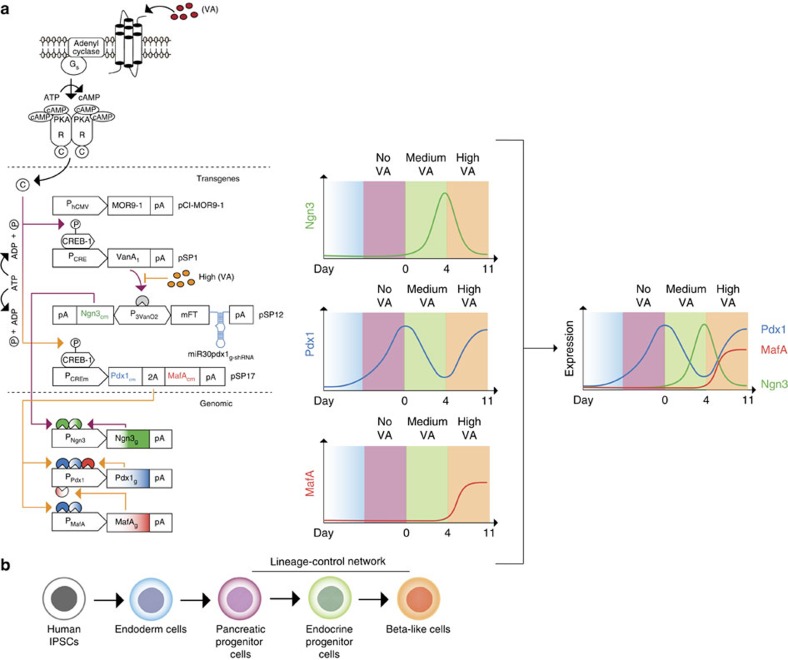
Synthetic lineage-control network programming differential expression dynamics of pancreatic transcription factors. (**a**) Schematic of the synthetic lineage-control network. The constitutively expressed, vanillic acid-sensitive olfactory G protein-coupled receptor MOR9-1 (pCI-MOR9-1; P_hCMV_-MOR9-1-pA) senses extracellular vanillic acid levels and triggers a synthetic signalling cascade, inducing P_CRE_-driven expression of the transcription factor VanA_1_ (pSP1, P_CRE_-VanA_1_-pA). At medium vanillic acid concentrations (purple arrows), VanA_1_ binds and activates the bidirectional vanillic acid-responsive promoter P_3VanO2_ (pSP12, pA-Ngn3_cm_←P_3VanO2_→mFT-miR30Pdx1_g-shRNA_-pA), which drives the induction of codon-modified Neurogenin 3 (*Ngn3*_*cm*_) as well as the coexpression of both the blue-to-red medium fluorescent timer (mFT) for precise visualization of the unit's expression dynamics and miR30pdx1_g-shRNA_ (a small hairpin RNA programming the exclusive destruction of genomic pancreatic and duodenal homeobox 1 (*Pdx1*_*g*_) transcripts). Consequently, Ngn3_cm_ levels switch from low to high (OFF-to-ON), and Pdx1_g_ levels toggle from high to low (ON-to-OFF). In addition, Ngn3_cm_ triggers the transcription of *Ngn3*_*g*_ from its genomic promoter, which initiates a positive-feedback loop. At high vanillic acid levels (orange arrows), VanA_1_ is inactivated, and both *Ngn3*_*cm*_ and miR30pdx1_g-shRNA_ are shut down. At the same time, the MOR9-1-driven signalling cascade induces the modified high-tightness and lower-sensitivity P_CREm_ promoter that drives the co-cistronic expression of the codon-modified variants of *Pdx1* (*Pdx1*_*cm*_) and V-maf musculoaponeurotic fibrosarcoma oncogene homologue A (*MafA*_*cm*_; pSP17, P_CREm_-Pdx1_cm_-2A-MafA_cm_-pA). Consequently, Pdx1_cm_ and MafA_cm_ become fully induced. As Pdx1_cm_ expression ramps up, it initiates a positive-feedback loop by inducing the genomic counterparts *Pdx1*_*g*_ and *MafA*_*g*_. Importantly, *Pdx1*_*cm*_ levels are not affected by miR30Pdx1_g-shRNA_ because the latter is specific for genomic *Pdx1*_*g*_ transcripts and because the positive feedback loop-mediated amplification of Pdx1_g_ expression becomes active only after the shutdown of miR30Pdx1_g-shRNA_. Overall, the synthetic lineage-control network provides vanillic acid-programmable, transient, mutually exclusive expression switches for Ngn3 (OFF-ON-OFF) and Pdx1 (ON-OFF-ON) as well as the concomitant induction of MafA (OFF-ON) expression, which can be followed in real time (Supplementary Movies 1 and 2). (**b**) Schematic illustrating the individual differentiation steps from human IPSCs towards beta-like cells. The colours match the cell phenotypes reached during the individual differentiation stages programmed by the lineage-control network shown in **a**.

For the design of the vanillic acid-programmable synthetic lineage-control network, constitutive MOR9-1 expression and P_CRE_-driven VanA_1_ expression were combined with **pSP12** (pA_SV40_-Ngn3_cm_←P_3VanO2_→mFT-miR30Pdx1_g-shRNA_-pA_SV40_) for endocrine specification and **pSP17** (P_CREm_-Pdx1_cm_-2A-MafA_cm_-pA_SV40_) for maturation of developing beta-cells (Fig. 2a,b). The **pSP12**-encoded expression unit enables the VanA_1_-controlled induction of the optimized bidirectional vanillic acid-responsive promoter (P_3VanO2_) that drives expression of a codon-modified *Ngn3*_*cm*_, the nucleic acid sequence of which is distinct from its genomic counterpart (*Ngn3*_*g*_) to allow for quantitative reverse transcription–PCR (qRT–PCR)-based discrimination. In the opposite direction, P_3VanO2_ transcribes miR30Pdx1_g-shRNA_, which exclusively targets genomic *Pdx1* (*Pdx1*_*g*_) transcripts for RNA interference-based destruction and is linked to the production of a blue-to-red medium fluorescent timer^40^ (mFT) for precise visualization of the unit's expression dynamics *in situ.* **pSP17** contains a dicistronic expression unit in which the modified high-tightness and lower-sensitivity P_CREm_ promoter (see below) drives co-cistronic expression of *Pdx1*_*cm*_ and *MafA*_*cm*_, which are codon-modified versions producing native transcription factors that specifically differ from their genomic counterparts (*Pdx1*_*g*_, *MafA*_*g*_) in their nucleic acid sequence. After individual validation of the vanillic acid-controlled expression and functionality of all network components (Supplementary Figs 2–9), the lineage-control network was ready to be transfected into hIPSC-derived pancreatic progenitor cells. These cells are characterized by high expression of Pdx1_g_ and Nkx6.1 levels and the absence of Ngn3_g_ and MafA_g_ production^32^,^33^,^34^ (day 0: Supplementary Figs 10–16).

**Figure 3:**
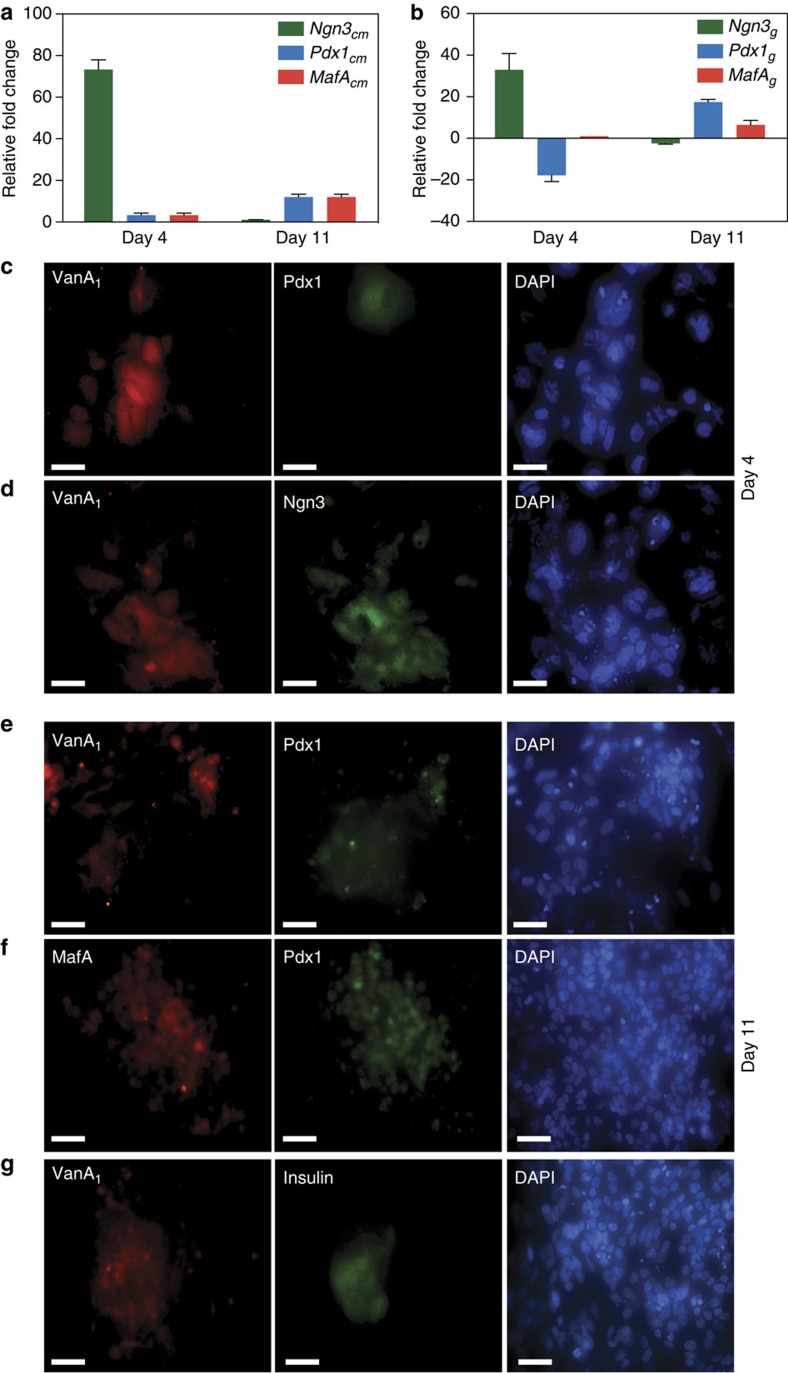
Dynamics of the lineage-control network. (**a**,**b**) Quantitative RT–PCR-based expression profiling of the pancreatic transcription factors *Ngn3*_*cm/g*_, *Pdx1*_*cm/g*_ and *MafA*_*cm/g*_ in hIPSC-derived pancreatic progenitor cells containing the synthetic lineage-control network at days 4 and 11. Data are the means±s.d. of triplicate experiments (*n*=9). (**c**–**g**) Immunocytochemistry of pancreatic transcription factors Ngn3_cm/g_, Pdx1_cm/g_ and MafA_cm/g_ in hIPSC-derived pancreatic progenitor cells containing the synthetic lineage-control network at days 4 and 11. hIPSC-derived pancreatic progenitor cells were co-transfected with the lineage-control vectors pCI-MOR9-1 (P_hCMV_-MOR9-1-pA), pSP1 (P_CRE_-VanA_1_-pA), pSP12 (pA-Ngn3_cm_←P_3VanO2_→mFT-miR30Pdx1_g-shRNA_-pA) and pSP17 (P_CREm_-Pdx1_cm_-2A-MafA_cm_) and immunocytochemically stained for (**c**) VanA_1_ and Pdx1 (day 4), (**d**) VanA_1_ and Ngn3 (day 4), (**e**) VanA_1_ and Pdx1 (day 11), (**f**) MafA and Pdx1 (day 11) as well as (**g**) VanA_1_ and insulin (C-peptide) (day 11). The cells staining positive for VanA_1_ are containing the lineage-control network. DAPI, 4′,6-diamidino-2-phenylindole. Scale bar, 100 μm.

Following the co-transfection of **pCI-MOR9-1** (P_hCMV_-MOR9-1-pA_SV40_), **pSP1** (P_CRE_-VanA_1_-pA_SV40_), **pSP12** (pA_SV40_-Ngn3_cm_←P_3VanO2_→mFT-miR30Pdx1_g-shRNA_-pA_SV40_) and **pSP17** (P_**CREm**_-Pdx1_cm_-2A-MafA_cm_-pA_SV40_) into hIPSC-derived pancreatic progenitor cells, the synthetic lineage-control network should override random endogenous differentiation activities and execute the pancreatic beta-cell-specific differentiation programme in a vanillic acid remote-controlled manner. To confirm that the lineage-control network operates as programmed, we cultivated network-containing and **pEGFP-N1**-transfected (negative-control) cells for 4 days at medium (2 μM) and then 7 days at high (400 μM) vanillic acid concentrations and profiled the differential expression dynamics of all of the network components and their genomic counterparts as well as the interrelated transcription factors and hormones in both whole populations and individual cells at days 0, 4, 11 and 14 (Figs 2 and 3 and Supplementary Figs 11–17).

### Validation of the lineage-control dynamics

The aforementioned detailed analysis demonstrated that the following three expression protocols were simultaneously executed:

Positive band-pass Ngn3 expression profile (OFF-ON-OFF): medium vanillic acid concentrations trigger the MOR9-1-based designer cascade and induce P_CRE_-driven VanA_1_ expression. Because VanA_1_ remains fully active at medium vanillic acid levels, it transactivates the bidirectional promoter P_3VanO2_ and induces *Ngn3*_cm_ expression (Fig. 3a). Ngn3_cm_ triggers the transcription of *Ngn3*_g_ from its genomic promoter, which initiates a positive-feedback loop^8^, resulting in high-level expression of Ngn3_g_ (OFF-ON; Fig. 3a–d and Supplementary Fig. 17) as well as its target genes *Pax4* (paired box gene 4) and the notch-signalling components *Hes1* (hairy and enhancer of split-1) and *Dll1* (delta-like 1), which manage lateral inhibition (a developmental pathway suppressing similar differentiation of neighbouring cells^8^,^41^; day 4; Supplementary Fig. 17). *NeuroD1* (neurogenic differentiation factor 1) expression levels in network-transfected cells remained identical to randomly differentiating cells (days 4; Supplementary Fig. 17). After switching the cells to high vanillic acid concentrations, VanA_1_ was released from P_3VanO2_ and Ngn3_cm_, and Ngn3_g_ expression halted, which resulted in an overall decrease of this transcription factor (ON-OFF; day 11; Fig. 3a,b).

Negative band-pass/band-stop filter Pdx1 expression profile (ON-OFF-ON): Following the MOR9-1-mediated activation of P_CRE_-driven VanA_1_ expression at medium vanillic acid concentrations, VanA_1_ co-induces the P_3VanO2_-driven expression of Ngn3_cm_ and miR30Pdx1_g-shRNA_. As Ngn3_cm_ and miR30Pdx1_g-shRNA_ levels increase, miR30Pdx1_g-shRNA_ programmes the exclusive destruction of genomic *Pdx1* (*Pdx1*_*g*_) transcripts, which results in a reduction of Pdx1_g_ levels (ON-OFF; Fig. 3b). Because P_CREm_-driven *Pdx1*_*cm*_*-2A-MafA*_*cm*_ transcription is silent at medium vanillic acid concentrations, the overall cellular Pdx1_cm/g_ content remains low (day 4; Fig. 3a–c). At high vanillic acid concentrations, where VanA_1_ is inactivated and P_3VanO2_-driven miR30Pdx1_g-shRNA_ expression is shut down, P_CREm_-mediated Pdx1_cm_ expression ramps up and initiates a positive-feedback loop by inducing *Pdx1*_*g*_^42^ as well *MafA*_*g*_^43^ (see below) from their genomic promoters, which results in sustained high-level Pdx1_g_ and MafA_g_ expression (OFF-ON; day 11; Fig. 3b,e–g). Importantly, *Pdx1*_*cm*_ levels are not affected by miR30Pdx1_g-shRNA_, as it is specific for genomic *Pdx1*_*g*_ transcripts, and the positive-feedback loop-mediated amplification of *Pdx1*_*g*_ expression becomes active only after the shutdown of miR30Pdx1_g-shRNA_ (Supplementary Fig. 18). The transition between miR30Pdx1_g-shRNA_-mediated knockdown and P_CREm_-driven Pdx1_cm_ ramp-up requires a time delay in the production of both components that results from the combination of the higher vanillic acid sensitivity and induction kinetics of P_CRE_ compared with P_CREm_ (Supplementary Fig. 7; see below) and the fact that miR30Pdx1_g-shRNA_ is driven by a two-level cascade (P_CRE_ inducing VanA_1_; VanA_1_ activating P_3VanO2_), which then leads to feed-forward loop-type signal amplification and increased expression kinetics of miR30Pdx1_g-shRNA_ compared with Pdx1_cm_ (Supplementary Fig. 18). The time delay and correlating expression dynamics of Ngn3_cm_, miR30Pdx1_g-shRNA_ and Pdx1_cm_ can be visualized in real time because miR30Pdx1_g-shRNA_ is linked to the expression of the blue-to-red mFT 40 and because the P_CREm_-mediated dicistronic expression of *Pdx1*_*cm*_*-2A-MafA*_*cm*_ can be linked to an isogenic P_CREm_-driven enhanced yellow fluorescent protein (EYFP) expression plug-in (**pSP24**; P_CREm_-EYFP-pA_SV40_). During the co-induction of Ngn3_cm_ and miR30Pdx1_g-shRNA_ as well as miR30Pdx1_g-shRNA_-mediated *Pdx1*_*g*_ knockdown at medium vanillic acid concentrations, the cells first fluoresce in blue and then simultaneously in blue and red as the mFT matures (Supplementary Movie 1). After switching the culture to high vanillic acid and repressing P_3VanO2_-driven Ngn3_cm_, miR30Pdx1_g-shRNA_ and mFT *de novo* synthesis, all cells contain fully matured mFT and exclusively fluoresce in red (Supplementary Movie 2). After the transition to P_CREm_-mediated Pdx1_cm_ and MafA_cm_ production, the cells express EYFP and fluoresce in yellow (Supplementary Movie 2).

MafA induction profile (OFF-ON): Because P_CREm_ has been optimized for tightness and decreased vanillic acid sensitivity^44^, MafA_cm_ is repressed at medium vanillic acid concentrations, as is required for the early phase of the differentiation process^45^ when Ngn3_cm_ is induced and Pdx1_g_ is repressed (day 4; Fig. 3a,b). At high vanillic acid levels, *MafA*_*cm*_ is co-cistronically expressed with *Pdx1*_*cm*_ by P_CREm_; therefore, the time-delayed production onset of MafA_cm_ matches that of Pdx1_cm_ (OFF-ON; Fig. 3a,b). MafA_cm_ triggers a positive-feedback loop by activating *Pdx1*_*g*_ expression^46^ from its genomic promoter, which results in sustained high-level MafA_g_ and Pdx1_g_ expression that compensates for the initial lower-level induction by P_CREm_ (day 11; Fig. 3a,b,e–g).

### Validation of beta-like cells programmed by the network

**Figure 4:**
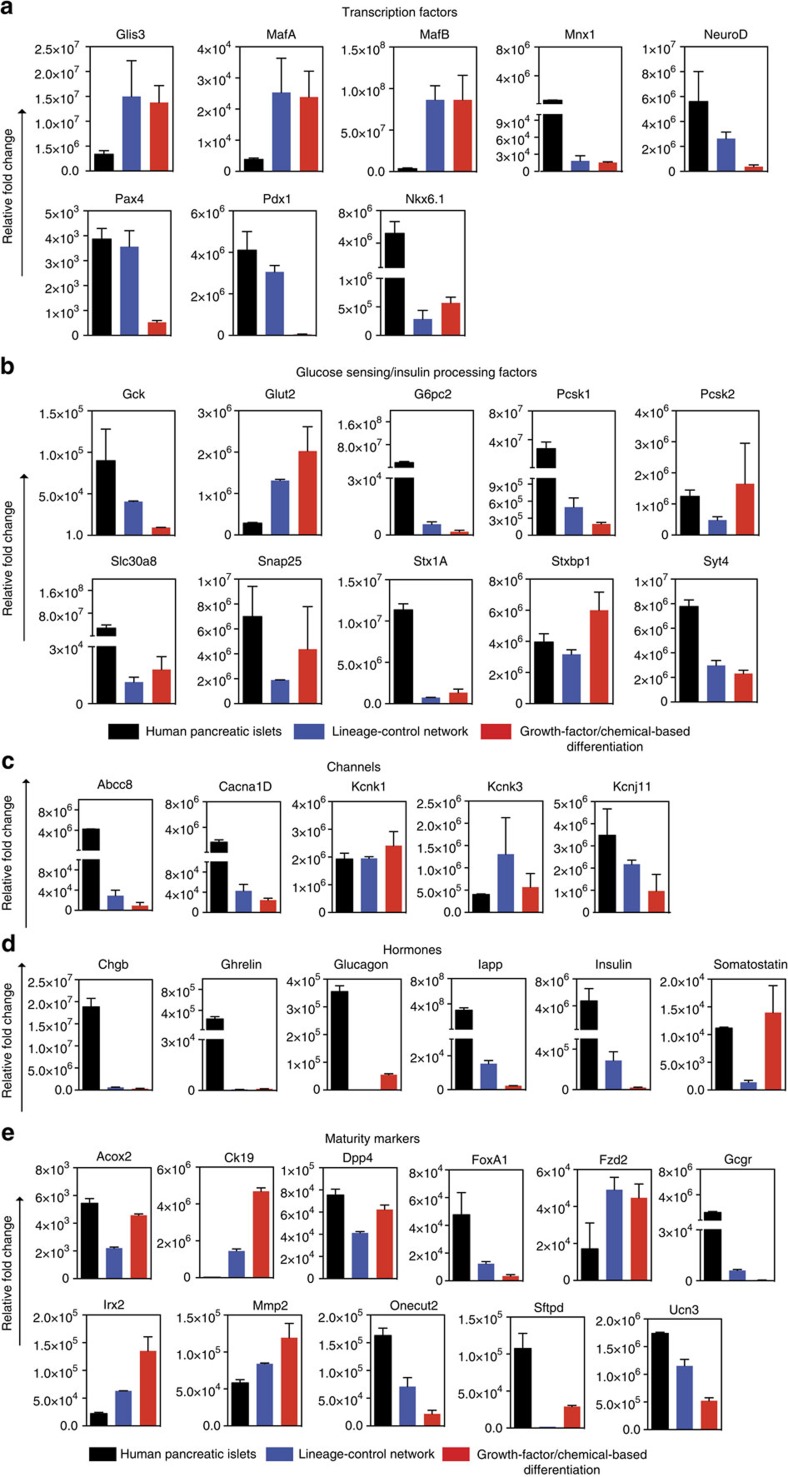
Gene expression profiling of beta-like cells programmed by the synthetic lineage-control network. Quantitative RT–PCR-based expression profiling of the following genes in glucose-sensitive insulin-secreting beta-like cells differentiated by the lineage-control network: (**a**) the key pancreatic beta-cell-specific transcription factors *Glis3, MafA, MafB, Mnx1, NeuroD, Pax4, Pdx1* and *Nkx6.1*, (**b**) the glucose- and insulin-processing factors *Gck, Glut2, G6pc2, Pcsk1, Pcsk2, Slc30a8, Snap25, Stx1A, Stxbp1, Syt4*, (**c**) the channels essential for the secretion of insulin such as *Abcc8, Cacna1D, Kcnk1/3* and *Kcnj11* and (**d**) the human islet peptide hormones *Chgb, Ghrelin, Glucagon, Iapp, Insulin* and *Somatostatin*, and (**e**) immature as well as mature human pancreatic beta-cell markers *Acox2, Ck19, Dpp4, FoxA1, Fzd2, Gcgr, Irx2, Mmp2, Onecut2, Sftpd* and *Ucn3*. The transcript levels were profiled at day 11 relative to hIPSCs and normalized to glyceraldehyde 3-phosphate dehydrogenase (GAPDH). Data are the means±s.d. of triplicate experiments (*n*=9). Abcc8, ATP-binding cassette transporter sub-family C member 8; Acox2, Acyl-CoA oxidase 2; Cacna1D, voltage-dependent, L-type alpha 1D subunit; Chgb, chromogranin B; Ck19, cytokeratin-19; Dpp4, dipeptidyl-peptidase 4; FoxA1, forkhead box protein A1; Fzd2, frizzled 2; Gcgr, glucagon receptor; Gck, glucokinase; Glis3, glis family zinc finger 3; Glut2, glucose transporter 2; G6pc2, glucose-6-phosphatase 2; Iapp, islet amyloid polypeptide; Irx2, iroquois homeobox 2; Kcnk1/3, potassium channel, subfamily K, member 1/3; Kcnj11, potassium inwardly-rectifying channel, subfamily J, member 11; MafA/B, V-maf musculoaponeurotic fibrosarcoma oncogene homologue A/B; Mmp2, matrix metalloproteinase 2; Mnx1, motor neuron and pancreas homeobox 1; NeuroD1, neurogenic differentiation factor 1; Nkx6.1, NK6 homeobox 1; Onecut2, onecut homeobox 2; Pax4, paired box gene 4; Pcsk1/2, proprotein convertase 1/2; Sftpd, surfactant protein D; Pdx1, pancreatic and duodenal homeobox 1; Slc30a8, solute carrier family 30, member 8; Snap25, synaptosomal-associated protein; Stx1A (Syntaxin-1A), Stxbp1, syntaxin binding protein 1; Syt4 synaptotagmin-4; Ucn3, urocortin 3.

To validate the beta-like cells genetically programmed from hIPSC-derived pancreatic progenitor cells using the synthetic lineage-control network, we compared their phenotype to cells produced using the growth-factor/chemical-based differentiation technique (Supplementary Figs 1 and 19) as well as to human pancreatic islets. Following individual execution of the two differentiation programmes (synthetic lineage-control network and growth-factor/chemical-based protocol; day 11), insulin-secreting beta-like cells were isolated by flow cytometry and profiled by quantitative RT–PCR for the expression levels of key beta-cell-specific markers relative to human pancreatic islets^32^,^33^,^34^,^47^,^48^,^49^ (Fig. 4).

**Figure 5:**
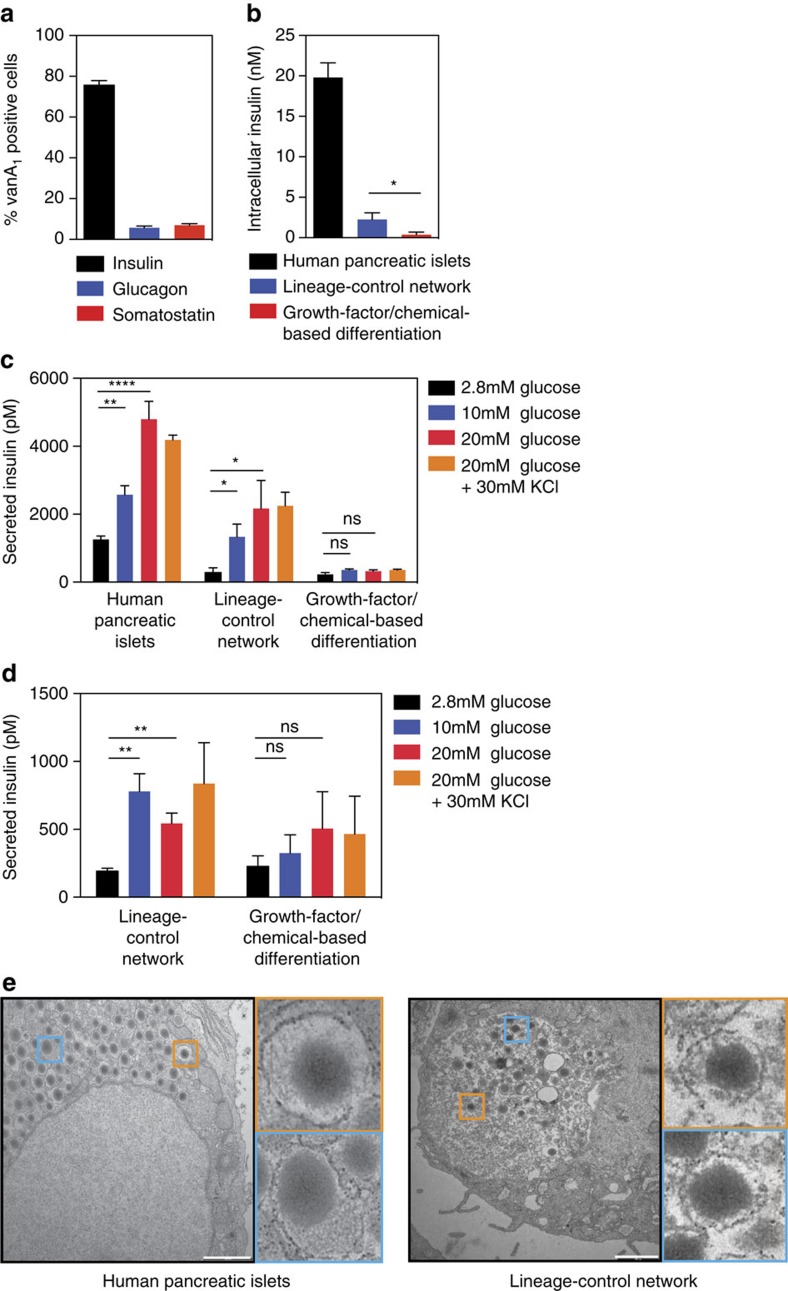
Characterization of glucose-sensitive insulin-secreting beta-like cells programmed by the synthetic lineage-control network. (**a**) Quantitative analysis of lineage-controlled beta-like cells co-stained for VanA_1_ and either insulin (C-peptide), glucagon or somatostatin. The cells staining positive for VanA_1_ are containing the lineage-control network. Data are the means±s.d. (*n*=3). (**b**,**c**) Human pancreatic islets and beta-like cells produced by programming hIPSC-derived pancreatic progenitor cells using the synthetic lineage-control network or the growth-factor/chemical-based differentiation technique were exposed to low (2.8 mM), medium (10 mM), high (20 mM) glucose as well as high glucose and potassium chloride (30 mM) before intracellular (**b**) and secreted (**c**) insulin (C-peptide) levels were profiled using ELISA. Data are the means±s.d. of duplicate experiments (*n*=6). Statistics by Student's *t*-test; *P<0.05, **P<0.005, ****P<0.0001; ns, not significant. (**d**) Beta-like cells produced by programming hIPSC-derived pancreatic progenitor cells using the synthetic lineage-control network or the growth-factor/chemical-based differentiation technique and cultivated for 4 weeks were exposed to low (2.8 mM), medium (10 mM), high (20 mM) glucose as well as high glucose and potassium chloride (30 mM) before secreted insulin (C-peptide) levels were profiled using ELISA. Data are the means±s.d. of duplicate experiments (*n*=6). Statistics by Student's *t*-test; **P<0.005; ns, not significant. (**e**) Transmission-electron micrographs of human pancreatic islets and lineage-controlled beta-like cells. Scale bars, 1 μm (human pancreatic islets) and 2 μm (beta-like cells differentiated by the synthetic lineage-control network).

Compared with human pancreatic islets, the beta-like cells produced by the synthetic lineage-control programme showed similar levels of (i) the transcription factors *MafA*, *NeuroD*, *Pax4* and *Pdx1*, whereas *NeuroD*, *Pax4* and *Pdx1* were expressed at lower levels in beta-like cells produced by the growth-factor/chemical-based differentiation technique (Fig. 4a). (ii) Likewise, the glucose-processing factors such as *Gck* (glucokinase) and *G6pc2* (glucose-6-phosphatase 2) as well as the insulin-processing factor *Pcsk1* (prohormone convertase 1) were expressed at higher levels in beta-like cells produced using the lineage-control network compared with those resulting from the growth-factor/chemical-based differentiation technique (Fig. 4b). (iii) The expression levels of channels essential for the secretion of insulin were comparable between beta-like cells produced by either differentiation strategy (Fig. 4c). (iv) *Insulin* expression was much higher in lineage-controlled than growth-factor/chemical-differentiated beta-like cells. Yet, *insulin* expression of lineage-controlled beta-like cells was not quite reaching the levels of human pancreatic islets (Fig. 4d). The expression of *somatostatin* and *glucagon* was substantially lower in lineage-controlled than growth-factor/chemical-differentiated beta-like cells (Fig. 4d). (v) Markers specific for immature beta-cells such as *Ck19* (Cytokeratin 19), *Irx2* (Iroquois homeobox 2) and *Acox2* (Acyl-CoA oxidase 2) were expressed at lower levels, whereas markers specific for mature beta-cells such as *FoxA1* (Forkhead box A1 (ref. 50) and *Gcgr* (Glucagon receptor) were expressed at a higher levels in lineage- than growth-factor/chemical-controlled beta-like cells (Fig. 4e). (vi) Flow-cytometric analysis of insulin (C-peptide), glucagon and somatostatin revealed that most lineage-controlled beta-like cells stained positive for the lineage-control network component VanA_1_ as well as insulin (C-peptide), whereas only very few VanA_1_-containing cells co-stained for glucagon and somatostatin (Fig. 5a and Supplementary Fig. 20a). These data confirmed that over 75% of the hIPSC-derived pancreatic progenitors transfected with the synthetic lineage-control network indeed differentiated into glucose-sensitive insulin-secreting beta-like cells (Fig. 5a and Supplementary Fig. 20a). By contrast, the growth-factor/chemical-based differentiation technique only produced 26% of insulin-secreting beta-like cells (Supplementary Fig. 20b,c). (vii) In addition, the intracellular insulin (C-peptide) content was substantially higher in lineage-controlled beta-like cells compared with those resulting from the growth-factor/chemical-based differentiation technique, albeit lower than in human pancreatic islets (Fig. 5b). (viii) Most importantly, the dynamics of glucose-stimulated insulin release of lineage-controlled beta-like cells was similar to that of human pancreatic islets, whereas beta-like cells differentiated by the growth factor/chemical-based differentiation technique showed poor glucose-stimulated insulin release (Fig. 5c). In addition, the lineage-controlled beta-like cells maintained glucose-stimulated insulin release during extended culture periods of up to 4 weeks (Fig. 5d).

Overall, all of these characteristics corroborate our finding that the synthetic lineage-control network is able to programme the cell fate of hIPSC-derived pancreatic progenitor cells and specifically differentiate them into glucose-sensitive insulin-secreting pancreatic beta-like cells. Indeed, electron micrographs of these lineage-controlled beta-like cells show the typical insulin-storage vesicles that are found in mature pancreatic beta cells (Fig. 5e).

## Discussion

Multicellular organisms, including humans, consist of a highly structured assembly of a multitude of specialized cell phenotypes that originate from the same zygote and have traversed a preprogrammed multifactorial developmental plan that orchestrates sequential differentiation steps with high precision in space and time^19^,^51^. Because of the complexity of terminally differentiated cells, the function of damaged tissues can for most medical indications only be restored via the transplantation of donor material, which is in chronically short supply^52^.

Despite significant progress in regenerative medicine and the availability of stem cells, the design of protocols that replicate natural differentiation programmes and provide fully functional cell mimetics remains challenging^29^,^53^. For example, efforts to generate beta-cells from human embryonic stem cells (hESCs) have led to reliable protocols involving the sequential administration of growth factors (activin A, bone morphogenetic protein 4 (BMP-4), basic fibroblast growth factor (bFGF), FGF-10, Noggin, vascular endothelial growth factor (VEGF) and Wnt3A) and small-molecule compounds (cyclopamine, forskolin, indolactam V, IDE1, IDE2, nicotinamide, retinoic acid, SB−431542 and γ-secretase inhibitor) that modulate differentiation-specific signalling pathways^31^,^54^,^55^. *In vitro* differentiation of hESC-derived pancreatic progenitor cells into beta-like cells is more challenging and has been achieved recently by a complex media formulation with chemicals and growth factors^32^,^33^,^34^.

hIPSCs have become a promising alternative to hESCs; however, their use remains restricted in many countries^56^. Most hIPSCs used for directed differentiation studies were derived from a juvenescent cell source that is expected to show a higher degree of differentiation potential compared with older donors that typically have a higher need for medical interventions^37^,^57^,^58^. We previously succeeded in producing mRNA-reprogrammed hIPSCs from adipose tissue-derived mesenchymal stem cells of a 50-year-old donor, demonstrating that the reprogramming of cells from a donor of advanced age is possible in principle^59^.

Recent studies applying similar hESC-based differentiation protocols to hIPSCs have produced cells that release insulin in response to high glucose^32^,^33^,^34^. This observation suggests that functional beta-like cells can eventually be derived from hIPSCs^32^,^33^. In our hands, the growth-factor/chemical-based technique for differentiating human IPSCs resulted in beta-like cells with poor glucose responsiveness. Recent studies have revealed significant variability in the lineage specification propensity of different hIPSC lines^35^,^60^ and substantial differences in the expression profiles of key transcription factors in hIPSC-derived beta-like cells^33^. Therefore, the growth-factor/chemical-based protocols may require further optimization and need to be customized for specific hIPSC lines^35^. Synthetic lineage-control networks providing precise dynamic control of transcription factor expression may overcome the challenges associated with the programming of beta-like cells from different hIPSC lines.

Rather than exposing hIPSCs to a refined compound cocktail that triggers the desired differentiation in a fraction of the stem cell population, we chose to design a synthetic lineage-control network to enable single input-programmable differentiation of hIPSC-derived pancreatic progenitor cells into glucose-sensitive insulin-secreting beta-like cells. In contrast with the use of growth-factor/chemical-based cocktails, synthetic lineage-control networks are expected to (i) be more economical because of *in situ* production of the required transcription factors, (ii) enable simultaneous control of ectopic and chromosomally encoded transcription factor variants, (iii) tap into endogenous pathways and not be limited to cell-surface input, (iv) display improved reversibility that is not dependent on the removal of exogenous growth factors via culture media replacement, (v) provide lateral inhibition, thereby reducing the random differentiation of neighbouring cells and (vi) enable trigger-programmable and (vii) precise differential transcription factor expression switches.

The synthetic lineage-control network that precisely replicates the endogenous relative expression dynamics of the transcription factors Pdx-1, Ngn3 and MafA required the design of a new network topology that interconnects a synthetic signalling cascade and a gene switch with differential and opposing sensitivity to the food additive vanillic acid. This differentiation device provides different band-pass filter, time-delay and feed-forward amplifier topologies that interface with endogenous positive-feedback loops to orchestrate the timely expression and repression of heterologous and chromosomally encoded Ngn3, Pdx1 and MafA variants. The temporary nature of the engineering intervention, which consists of transient transfection of the genetic lineage-control components in the absence of any selection, is expected to avoid stable modification of host chromosomes and alleviate potential safety concerns. In addition, the resulting beta-cell mass could be encapsulated inside vascularized microcontainers^28^, a proven containment strategy in prototypic cell-based therapies currently being tested in animal models of prominent human diseases^14^,^15^,^16^,^61^,^62^ as well as in human clinical trials^28^.

The hIPSC-derived beta-like cells resulting from this trigger-induced synthetic lineage-control network exhibited glucose-stimulated insulin-release dynamics and capacity matching the human physiological range and transcriptional profiling, flow cytometric analysis and electron microscopy corroborated the lineage-controlled stem cells reached a mature beta-cell phenotype. In principle, the combination of hIPSCs derived from the adipose tissue of a 50-year-old donor^59^ with a synthetic lineage-control network programming glucose-sensitive insulin-secreting beta-like cells closes the design cycle of regenerative medicine^63^. However, hIPSCs that are derived from T1DM patients, differentiated into beta-like cells and transplanted back into the donor would still be targeted by the immune system, as demonstrated in the transplantation of segmental pancreatic grafts from identical twins^64^. Therefore, any beta-cell-replacement therapy will require complementary modulation of the immune system either via drugs^30^,^65^, engineering or cell-based approaches^66^,^67^ or packaging inside vascularizing, semi-permeable immunoprotective microcontainers^28^.

Capitalizing on the design principles of synthetic biology, we have successfully constructed and validated a synthetic lineage-control network that replicates the differential expression dynamics of critical transcription factors and mimicks the native differentiation pathway to programme hIPSC-derived pancreatic progenitor cells into glucose-sensitive insulin-secreting beta-like cells that compare with human pancreatic islets at a high level. The design of input-triggered synthetic lineage-control networks that execute a preprogrammed sequential differentiation agenda coordinating the timely induction and repression of multiple genes could provide a new impetus for the advancement of developmental biology and regenerative medicine.

## Methods

### Network components

Comprehensive design and construction details for all expression vectors are provided in Supplementary Table 1. Key plasmids are as follows: **pCI-MOR**_**9-1**_ encodes constitutively expressed vanillic acid receptor (**MOR9-1**; P_hCMV_-MOR9-1-pA_SV40_); **pSP1** encodes cAMP-responsive expression cassette for the vanillic acid-dependent transactivator (VanA_1_; P_CRE_-VanA_1_-pA_SV40_); **pSP12** contains a vanillic acid-responsive Ngn3_cm_, mFT and miR30Pdx1_g-shRNA_ expression unit (pA_SV40_←Ngn3_cm_-P_3VanO2_→mFT-miR30Pdx1_g-shRNA_-pA_SV40_) and **pSP17** contains a cAMP-responsive dicistronic Pdx1_cm_ and MafA_cm_ expression unit (P_CREm_-Pdx1_cm_-2A-MafA_cm_-pA_SV40_).

### Cell culture and transfection

Human mesenchymal stem cells transgenic for the catalytic subunit of human telomerase (hMSC-TERT^68^) were cultivated in DMEM (Invitrogen) supplemented with 10% (vol/vol) fetal calf serum (FCS; lot no. PE01026P, Bioconcept) and 1% (vol/vol) penicillin/streptomycin solution (Sigma-Aldrich) at 37 °C in a humidified atmosphere containing 5% CO_2_. The hIPSCs were derived from the adipose tissue of a 50-year-old donor^59^. The hIPSCs were cultivated in Geltrex-coated 12-well culture plates (Invitrogen) containing 1 ml mTeSR1 medium (STEMCELL Technologies). For serial passage, the colonies were enzymatically dissociated into cellular clumps (200–400 cells per clump) using 1 U ml^−1^ dispase (STEMCELL Technologies). For the transfection of hMSC-TERT, 2 × 10^5^ cells were seeded per well on a six-well plate 12 h before transfection and incubated for 24 h with 400 μl of a DNA-polyethyleneimine (PEI) mixture that was produced by incubating 6 μl PEI (PEI, <20,000 MW, Polysciences; stock solution: 1 mg ml^−1^ ddH_2_O, pH 7.2) with 2 μg of total DNA, vortexing for 5 s and incubating for 15 min at 22 °C. Before transfection, the hIPSCs-derived pancreatic progenitor cells were dissociated using 0.5 ml of StemPro Accutase Cell Dissociation Reagent (Invitrogen) and reseeded into 12-well plates. For the transfection, 3 μg of total DNA was transfected into cells cultivated in each well of a 12-well plate using Lipofectamine LTX Reagent with PLUS Reagent (Invitrogen). The plasmids (**pCI-MOR9-1**; **pSP1**; **pSP12**; **pSP17**) were co-transfected at a ratio of 1:1:1:1 for hIPSCs (1:0.01:1:1 for hMSC-TERT). The transfection efficiency was determined via FACS analysis using pEGFP-N1-transfected, randomly differentiating cells as a control (Supplementary Fig. 10). Randomly differentiating or lineage-controlled hIPSC-derived pancreatic progenitor cell populations (day 11) were prepared for transfection by dissociating the cells with 0.5 ml of StemPro Accutase Cell Dissociation Reagent (Invitrogen), seeded into the wells of a 12-well plate and cultivated for 4 h in 1 ml RPMI (Invitrogen) containing 10% FCS and supplemented with 10 μM Y-27632, 11 mM glucose, 400 μM vanillic acid (Sigma-Aldrich), 50 ng ml^−1^ exendin-4 (Tocris Bioscience), 10 mM nicotinamide (Sigma-Aldrich) and 40 μM β-mercaptoethanol. Subsequently, the culture medium was replaced by the same supplemented RPMI devoid of Y-27632 and the cells were transfected with 3 μg **pSP26** (P_hINS_-DsRed-Express-pA_SV40_) using Lipofectamine LTX with Plus Reagent (Invitrogen). For FACS-mediated single-cell sorting of the lineage- and growth-factor/chemical-controlled beta-like cells (day 11), hIPSC-derived pancreatic progenitor cells (day 0) were co-transfected with 3 μg of total DNA (**pCI-MOR9-1**, **pSP1**, **pSP12**, **pSP17**, **pEGFP-N1**, **pSP26**; for the lineage-control network; **pEGFP-N1**, **pSP26** for the growth-factor/chemical-based differentiation technique). The parental vector pcDNA3.1(+) was used as a filler plasmid when replacing functional components for control purposes. For the dose–response experiments, the transfected cells were trypsinized (200 μl trypsin, 5 min, 37 °C; PAN Biotech) and transferred into the wells of a 96-well plate (10^4^ cells per well) containing 100 μl DMEM supplemented with different concentrations of vanillic acid. SEAP expression was profiled after 48 h. To compare vanillic acid-triggered lineage control with independent expression control of the pancreatic transcription factors Ngn3, Pdx1, MafA by different antibiotic-responsive gene switches, we co-transfected hMSC-TERT with **pCI-MOR9-1**, **pSP1**, **pSP12** and **pSP17** for the lineage-control network, with **pWW35**, **pSP36**, **pMF156** and **pSP39** for macrolide-responsive Pdx1 and MafA (E_OFF_) as well as streptogramin-responsive Ngn3 expression (PIP_OFF_) or with **pWW35**, **pSP37**, **pMF156**, **pSP39**, **pTet-ON** and **pSP4** for macrolide-responsive Pdx1 (E_OFF_), streptogramin-responsive Ngn3 (PIP_OFF_) and tetracycline-responsive MafA (TET_ON_) expression, and incubated the cells in the presence of vanillic acid (2 μM medium concentration, 400 μM high concentration), erythromycin (1 μM; Sigma-Aldrich), doxycycline (1 μM; Sigma-Aldrich) or pristinamycin (Pyostacin, 1 μM; Sanofi-Aventis Inc.).

### Differentiation of hIPSCs

The hIPSCs were differentiated to pancreatic progenitor cells and beta-like cells using the growth-factor/chemical-based differentiation techniques described below^32^,^33^,^34^,^55^,^60^. Step 1 (endoderm cells): Human pluripotent stem cells (80–90% confluence) were washed with 1 × PBS and cultivated for 24 h in RPMI containing Activin A (100 ng ml^−1^) and Wnt3A (25 ng ml^−1^). Then, the cells were cultivated for another 48 h in RPMI containing Activin A (100 ng ml^−1^), bFGF (5 ng ml^−1^), BMP-4 (0.25 ng ml^−1^) and VEGF_165_ (10 ng ml^−1^). Subsequently, the cells were cultivated for another 48 h in serum-free differentiation (SFD) medium (75% Iscove's modified Dulbecco's medium, 25% Ham's F-12, 0.5 × N2 supplement, 0.5 × vitamin A-free B−27 serum-free supplement, 0.1% bovine serum albumin, ascorbic acid (50 μg ml^−1^), 1-thioglycerol (0.45 μM)) containing Activin A (100 ng ml^−1^), bFGF (5 ng ml^−1^), BMP-4 (0.25 ng ml^−1^) and VEGF_165_ (10 ng ml^−1^). Step 2 (primitive gut tube cells): The endoderm cells resulting from step 1 were cultivated for 72 h in SFD medium containing Wnt3A (3 ng ml^−1^), Noggin (50 ng ml^−1^) and FGF-10 (50 ng ml^−1^). Step 3 (pancreatic progenitor cells): The primitive gut tube cells resulting from step 2 were cultivated for 72 h in DMEM containing 1% (vol/vol) vitamin A-free B−27 serum-free supplement, ascorbic acid (50 μg ml^−1^), KAAD-cyclopamine (0.25 μM), retinoic acid (2 μM) and FGF-10 (50 ng ml^−1^). Step 4 (pancreatic progenitor cells): The pancreatic progenitor cells resulting from step 3 were cultivated for 72 h in DMEM containing 1% (vol/vol) vitamin A-free B−27 serum-free supplement, ascorbic acid (50 μg ml^−1^), retinoic acid (100 nM), FGF-7 (50 ng ml^−1^) and epidermal growth factor (EGF; 50 ng ml^−1^). Step 5 (endocrine progenitor cells): The pancreatic progenitor cells resulting from step 4 were treated for 4 h with Y-27632 (10 μM), dissociated for 5 min using 0.5 ml of StemPro Accutase and incubated for 72 h in suspension plates to facilitate the formation of aggregates in DMEM containing 1% (vol/vol) vitamin A-free B−27 serum-free supplement, ascorbic acid (50 μg ml^−1^), retinoic acid (50 nM), thyroid hormone T3 (1 μM), Alk5 inhibitor (10 μM), Noggin (50 ng ml^−1^), KAAD-cyclopamine (0.25 μM) and gamma secretase inhibitor (1 μM). Step 6 (maturing beta-like cells): The endocrine progenitor cells resulting from step 5 were cultivated for 72 h in DMEM containing 1% (vol/vol) vitamin A-free B−27 serum-free supplement, ascorbic acid (50 μg ml^−1^), retinoic acid (25 nM), thyroid hormone T3 (1 μM), Noggin (50 ng ml^−1^), Alk5 inhibitor (10 μM) and gamma secretase inhibitor (1 μM). Step 7 (beta-like cells): The maturing beta-like cells resulting from step 6 were cultivated for 5 days in low-glucose DMEM (5 mM glucose) supplemented with 10% (vol/vol) FCS and containing 1 × non-essential amino acid solution, thyroid hormone T3 (1 μM) and Alk5 inhibitor (10 μM). For the lineage-control network, 12-well suspension plates (Greiner Bio-one GmbH.) were used from day 0 onwards and hIPSC-derived endodermal cells were cultivated in noggin- and gamma-secretase inhibitor-free culture medium (to prevent premature endocrine differentiation before transfection of the lineage-control network) supplemented with epidermal growth factor (to trigger expression of Nkx6.1 in pancreatic progenitor cells). After dissociation and transfection of the pancreatic progenitor cells with the lineage-control network, the engineered cells spontaneously form aggregates and following addition of thyroid hormone T3 (triiodothyronine) and retinoic acid they differentiate into endocrine progenitor cells and mature into beta-like cells following addition of Alk5 inhibitor (Supplementary Fig. 1). Randomly differentiating pancreatic progenitor cells were cultivated in DMEM supplemented with 10% (vol/vol) KnockOut Serum Replacement (KOSR) for 4 days and then for 7 days in SFD supplemented with 10% (vol/vol) KOSR. Roswell Park Memorial Institute (RPMI) 1640 culture medium, Iscove's modified Dulbecco's medium, Ham's F12 nutrient mixture, Vitamin A-free B−27 serum-free supplement, N-2 supplement, human bFGF, KOSR, non-essential amino-acid solution, PBS and StemPro Accutase cell dissociation reagent were purchased from Invitrogen. Human BMP4, the sonic hedgehog antagonist KAAD-Cyclopamine, human Noggin, human EGF, human fibroblast growth factor 7/10 (FGF-7/FGF-10) and human VEGF_165_ were obtained from Miltenyi Biotec. Human Activin A, wingless-type MMTV integration site family member 3A (Wnt3A), vanillic acid, ascorbic acid, bovine serum albumin, 1-thioglycerol, thyroid hormone T3 and retinoic acid were purchased from Sigma-Aldrich. The gamma secretase inhibitor L-685,458 was obtained from Tocris Bioscience, Rock inhibitor Y-27632 was obtained from STEMCELL Technologies and Alk5 inhibitor was obtained from Enzo Lifesciences.

### Immunocytochemistry

At different time points of the differentiation (endoderm cells; pancreatic progenitor cells, endocrine progenitor cells; pancreatic beta-like cells; Supplementary Fig. 1), network-containing hIPSCs cultivated in 12-well plates were fixed with 4% (wt/vol) paraformaldehyde solution overnight at 4 °C. The fixed cells were permeabilized with 0.1% (vol/vol) Triton X-100 solution and then incubated with blocking solution (10% (vol/vol) goat serum in PBS) for 1 h at 37 °C followed by 4′,6-diamidino-2-phenylindole-mediated staining of the cell nuclei (Vectashield; Vector Labs). The samples were immunostained for 1 h at 37 °C using primary antibodies specific for Sox17 and Fox A2 (endoderm cells), Pdx1, Nkx6-1 (pancreatic progenitor cells), Ngn3, Pdx1 and VP16 (endocrine progenitor cells) and insulin (C-peptide), VP16, Pdx1, MafA, glucagon and somatostatin (pancreatic beta-like cells), washed three times in 0.1% Tween-20 and incubated for 1 h with appropriate fluorophore-conjugated secondary antibodies before immunofluorescence was visualized with a Nikon Eclipse Ti microscope (Nikon AG) using the appropriate excitation/emission wavelengths. The primary antibodies utilized in this study included mouse anti-Sox17 IgG (MAB19241, lot no. C614013; dilution 1:1,000; R&D Systems Europe), rabbit anti-FoxA2 IgG (AB4125, lot no. NMM1757191; dilution 1:1,000; Millipore), rabbit anti-VP16 IgG (ab4808, lot no. 830675; dilution 1:200; Abcam), mouse anti-Ngn3 IgG (F25A1B3, lot no. GR95038; dilution 1:100; Developmental Studies Hybridoma Bank), mouse anti-Pdx1 IgG (MAB2419, lot no. C267712; dilution 1:2,000; R&D Systems), rabbit anti-MafA IgG (sc-66958, lot no. C1609; dilution 1:100; Santa Cruz Biotechnology), rat anti-C-peptide IgG (GN-ID4, lot no. 10510; dilution 1:50; Developmental Studies Hybridoma Bank), goat anti-glucagon IgG (sc-7779, lot no. E2112; dilution 1:200; Santa Cruz Biotechnology), mouse anti-somatostatin IgG (sc-74556, lot no. J2708; dilution 1:200; Santa Cruz Biotechnology) and rat anti-Nkx6-1 IgG (F55A12-s, dilution 1:200; Developmental Studies Hybridoma Bank). The secondary antibodies utilized in this study included fluorescein (FITC)-conjugated goat anti-mouse IgG (AP124F, lot no. LV1688510; dilution 1:1,000), rhodamine-conjugated goat anti-rabbit IgG (AP307R, lot no. LV1441875; dilution 1:1,000) from Millipore Inc, donkey anti-goat IgG (sc-3860, lot no. G1712; dilution 1:400) and fluorescein (FITC)-conjugated chicken anti-rat IgG (sc-2991, lot no. K2912; dilution 1:400) from Santa Cruz Biotechnology.

### Analytical assays

The production of human placental SEAP was quantified in cell culture supernatants using a *p*-nitrophenylphosphate-based light absorbance time course^69^. Activity of *Gaussia princeps* Luciferase was quantified using a BioLux *Gaussia* Luciferase Assay Kit (New England Biolabs Inc.). The activity of *Photinus pyralis* firefly luciferase (FLuc) was quantified using a Luciferase Assay System (Promega).

### Quantitative RT–PCR

Total RNA of lineage-controlled hIPSC population, FACS-sorted hIPSC-derived beta-like cells, engineered hMSC-TERT and human islets was isolated using the ZR RNA MiniPrep kit (Zymo Research) and TURBO DNase (Invitrogen). qRT–PCR with total RNA was performed using SuperScript II Reverse Transcriptase (Invitrogen) and TaqMan Fast Advanced Mastermix (Invitrogen) with the corresponding TaqMan Gene Expression Assays (Supplementary Table 2) or SYBR Green PCR Mastermix with custom-designed primers (Supplementary Table 3). qRT–PCR with total RNA of FACS-sorted hIPSC-derived beta-like cells and human islets was performed using High-Capacity cDNA Reverse Transcription Kit (Invitrogen) and Taqman PreAmp Mastermix Kit (Invitrogen) with the corresponding TaqMan Gene Expression Assays (Supplementary Table 2). The Eppendorf Realplex Mastercycler (Eppendorf GmbH) was set to the following amplification parameters: 2 min at 50 °C, 20 s at 95 °C and 40 cycles of 1 s at 95 °C followed by 1 min at 60 °C. The relative threshold cycle (*C*_t_) was determined and normalized to the endogenous glyceraldehyde 3-phosphate dehydrogenase transcript. The fold change for each transcript relative to the control was calculated using the comparative *C*_t_ method^70^.

### FACS-mediated analysis and single-cell sorting

For FACS-mediated analysis and single-cell sorting of randomly differentiating and lineage-controlled hIPSC-derived beta-like cells (day 11), the cell populations was co-transfected with **pSP26** (P_hINS_-DsRed-Express-pA_SV40_) and the resulting beta-like cells were sorted (day 14) using a Becton Dickinson LSRII Fortessa flow cytometer (Becton Dickinson) equipped for DsRed (561 nm laser, 570 nm long-pass filter, 586/15 emission filter) detection. For FACS-mediated analysis and single-cell sorting of hIPSC-derived beta-like cells differentiated using the growth-factor/chemical-based differentiation technique (day 11), the respective treated population (day 0) was co-transfected with 3 μg of total DNA (**pCI-MOR9-1**, **pSP1**, **pSP12**, **pSP17**, **pEGFP-N1**, **pSP26**) for the lineage-control network; (**pEGFP-N1**, **pSP26**) for the growth-factor/chemical-based differentiation technique and the resulting beta-like cells were sorted using DsRed (561 nm laser, 570 nm long-pass filter, 586/15 emission filter) detection and EGFP (488 nm laser, 505 nm long-pass filter, 530/30 emission filter) detection while excluding dead cells and cell doublets. Total RNA extracted from the sorted beta-like cells was used for qRT–PCR. For FACS analysis, we used the same antibodies and dilutions as for immunostaining. In brief, cells were detached (1 × 10^6^ cells per ml) using 0.5 ml StemPro Accutase Cell Dissociation Reagent (Invitrogen) and fixed with 4% (wt/vol) paraformaldehyde in PBS overnight at 4 °C, permeabilized for 10 min with 0.1% (vol/vol) Triton X-100, washed once in 2 ml PBS and sequentially labelled for 1 h at 37 °C with specific primary and secondary antibodies, with a PBS washing step in between. The cell populations were analysed using a Becton Dickinson LSRII Fortessa flow cytometer (Becton Dickinson) equipped for EGFP (488 nm laser, 505 nm long-pass filter, 530/30 emission filter), DsRed (561 nm laser, 570 nm long-pass filter, 586/15 emission filter) or allophycocyanin (ACP, 633 nM laser, 670/14 emission filter) detection and set to exclude dead cells and cell doublets. Unstained cell populations were used as negative controls.

### Transmission electron microscopy

Human islets and beta-like cells differentiated using the lineage-control network (day 11) were dissociated into single cells using 0.5 ml of StemPro Accutase Cell Dissociation Reagent (Invitrogen) and prepared for electron-microscopic analysis using a standard procedure^34^. In brief, after embedding the cell pellets in EMbed 812 resin (Electron Microscopy Sciences), serial 70 nm sections were collected on formvar-coated copper slot grids (Electron Microscopy Sciences) and micrographs were taken using a FEI/Philips CM-10 transmission electron microscope (FEI/Philips) equipped with a 2k × 2k CCD Veleta camera (EMSIS, GmbH).

### Quantification of glucose responsiveness

Human islets and beta-like cells either differentiated by the synthetic lineage-control network or using the growth-factor/chemical-based differentiation technique (day 11) were washed in 0.25 ml Krebs-Ringer Bicarbonate Buffer (129 mM NaCl, 5 mM NaHCO_3_, 4.8 mM KCl, 1.2 mM KH_2_PO_4_, 1.2 mM MgSO_4_, 2.5 mM CaCl_2_, 10 mM HEPES, pH 7.4; Sigma-Aldrich) and incubated for 30 min in low-glucose (Sigma-Aldrich; 2.8 mM) Krebs-Ringer Bicarbonate Buffer supplemented with 100 μM 3-isobutyl-1-methylxanthine (Sigma-Aldrich). The culture was then switched to intermediate glucose (10 mM) for 30 min and then high-glucose (20 mM) Krebs-Ringer Bicarbonate Buffer for another 30 min. Thereafter, the culture was switched to high-glucose (20 mM) and KCl (30 mM)-supplemented Krebs-Ringer Bicarbonate Buffer for another 30 min. The intracellular (released via cell lysis for 5 min at 22 °C in M-PER protein lysis buffer; Thermo Scientific) and secreted isoforms of the connecting peptide (C-peptide) produced during proinsulin processing were quantified using the ultrasensitive human C-peptide ELISA (lot no. 21899; Mercodia). The values were normalized to the total intracellular protein content and the number of glucose-sensitive insulin-secreting cells using a Bio-Rad protein assay (Bio-Rad Laboratories). For the extended culture, beta-like cells (day 11) were continuously cultivated for 4 weeks in DMEM (Invitrogen) containing 10% (vol/vol) FCS, 5 mM glucose, 400 μM vanillic acid (Sigma-Aldrich), 1 μM thyroid hormone T3 (Sigma-Aldrich) and 10 μM Alk5 inhibitor before glucose-stimulated insulin release was quantified.

### Statistical analyses

All data represent the means±s.d. of three independent experiments with two or three samples per experiment. Group comparisons were analysed by the Student's *t*-test (cutoff of *P*<0.05) using GraphPad Prism 6 software (GraphPad Software Inc.).

## Additional information

**How to cite this article**: Saxena, P. *et al*. A programmable synthetic lineage-control network that differentiates human IPSCs into glucose-sensitive insulin-secreting beta-like cells. *Nat. Commun.* 7:11247 doi: 10.1038/ncomms11247 (2016).

## Supplementary Material

Supplementary Information Supplementary Figures 1-20, Supplementary Tables 1-3 and Supplementary References

Supplementary Movie 1 Time-lapse fluorescence microscopy of hMSC-TERT cotransfected with the lineagecontrol network vectors pCI-MOR9-1 (PhCMV-MOR9-1-pA), pSP1 (PCRE-VanA1-pA), pSP12 (pA-Ngn3cm←P3VanO2→mFTmiR30Pdx1g-shRNA-pA) and pSP24 (PCREm-EYFP-pA; visual plug-in for pSP17) and grown for 59h in the presence of medium (2μM) vanillic acid concentration.

Supplementary Movie 2 Time-lapse fluorescence microscopy of hMSC-TERT cotransfected with the lineagecontrol network vectors pCI-MOR9-1 (PhCMV-MOR9-1-pA), pSP1 (PCRE-VanA1-pA), pSP12 (pA-Ngn3cm←P3VanO2→mFTmiR30Pdx1g-shRNA-pA) and pSP24 (PCREm-EYFP-pA; visual plug-in for pSP17) and grown for 48h in the presence of high (400μM) vanillic acid concentration.
