# Supplementary material for: A programmable synthetic lineage-control network that differentiates human IPSCs into glucose-sensitive insulin-secreting beta-like cells
Source: Nat Commun. 2016 Apr 11;7:11247. doi: 10.1038/ncomms11247 (PMC4831023; doi:10.1038/ncomms11247)
Supplement: Supplementary Information — Supplementary Figures 1-20, Supplementary Tables 1-3 and Supplementary References [file ncomms11247-s1.pdf]

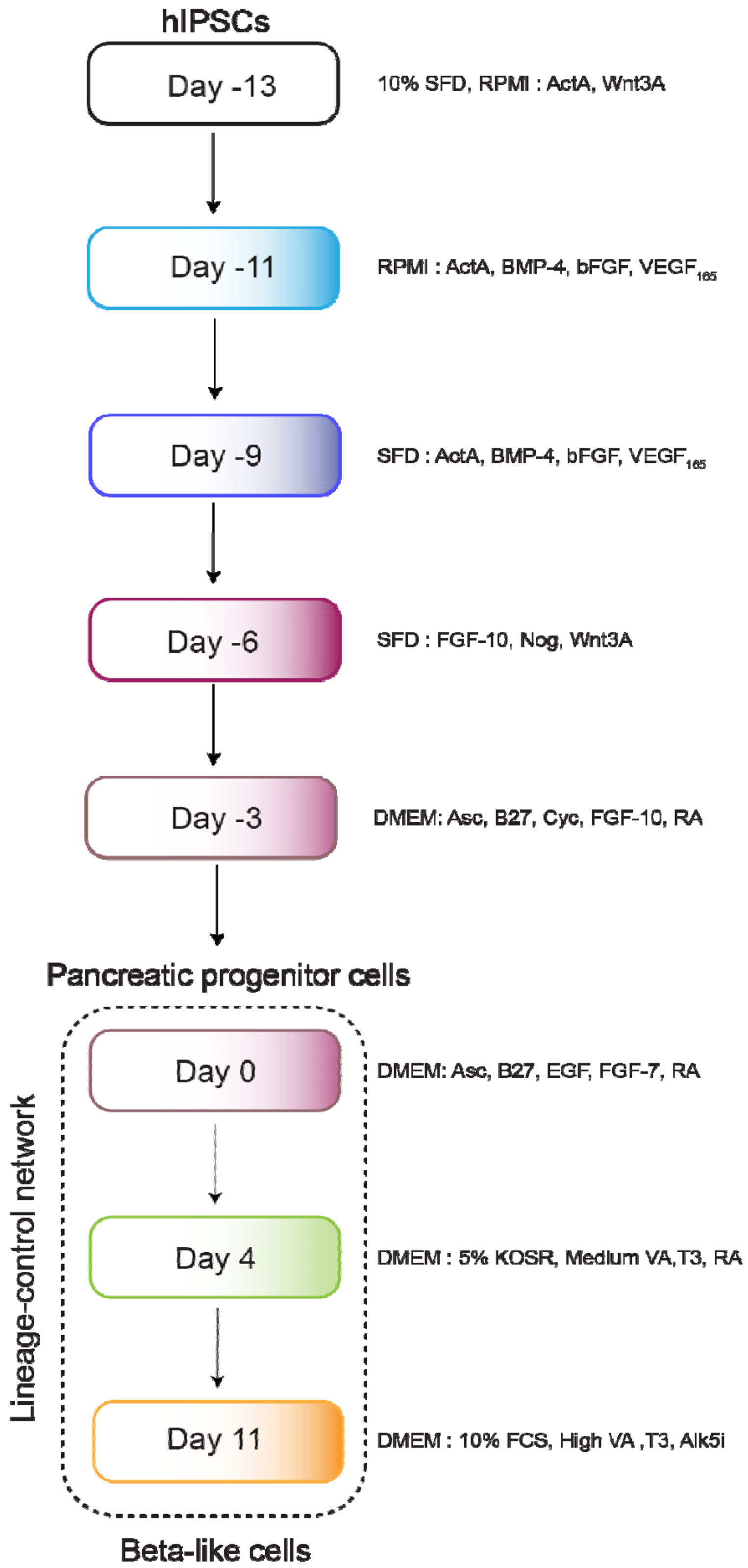
hIPSCs
Day -13
10% SFD, RPMI : ActA, Wnt3A
Day -11
RPMI : ActA, BMP-4, bFGF, VEGF165
Day -9
SFD : ActA, BMP-4, bFGF, VEGF165
Day -6
SFD : FGF-10, Nog, Wnt3A
Day -3
DMEM: Asc, B27, Cyc, FGF-10, RA
Pancreatic progenitor cells
Lineage-control network
Day 0
DMEM: Asc, B27, EGF, FGF-7, RA
Day 4
DMEM : 5% KOSR, Medium VA,T3, RA
Day 11
DMEM : 10% FCS, High VA ,T3, Alk5i
Beta-like cells

Supplementary Figure 1. Illustration of the differentiation protocol involving the synthetic lineage-control network to program human induced pluripotent stem cells (hIPSCs) into glucose-sensitive insulin-secreting beta-like cells. hIPSCs are differentiated for 13 days (Day -13 until Day 0) into pancreatic progenitor cells using a standard growth-factor cocktail. Transfection of expression vectors encoding the lineage-control network into hIPSC-derived pancreatic progenitor cells enables vanillic-acid-triggered induction of the lineage-control program production of glucose-sensitive insulin-secreting beta-cells (the color code matches with the cell phenotypes and network dynamics shown in Figure 2). Abbreviations: **ActA**: Activin A; **Alk5i**: Alk5 inhibitor II; **Asc**: Ascorbic acid; **bFGF**: Basic fibroblast growth factor; **BMP4**: Bone morphogenetic protein 4; **B27**: B27 supplement; **Cyc**: KAAD cyclopamine; **DMEM**: Dulbecco's modified eagle's medium; **EGF**: Epidermal growth factor; **FCS**: Fetal calf serum; **FGF7**: Fibroblast growth factor 10; **FGF10**: Fibroblast growth factor 10; **KOSR**: Knockout serum replacement, **Nog**: Noggin; **RA**: Retinoic acid; **RPMI**: Roswell park memorial institute medium; **SFD**: Serum free defined medium; **T3**: Triiodothyronine; **VA**: Vanillic acid; **$VEGF_{165}$**: Vascular endothelial growth factor; **Wnt3A**: Wingless-type MMTV integration site family, member 3A.

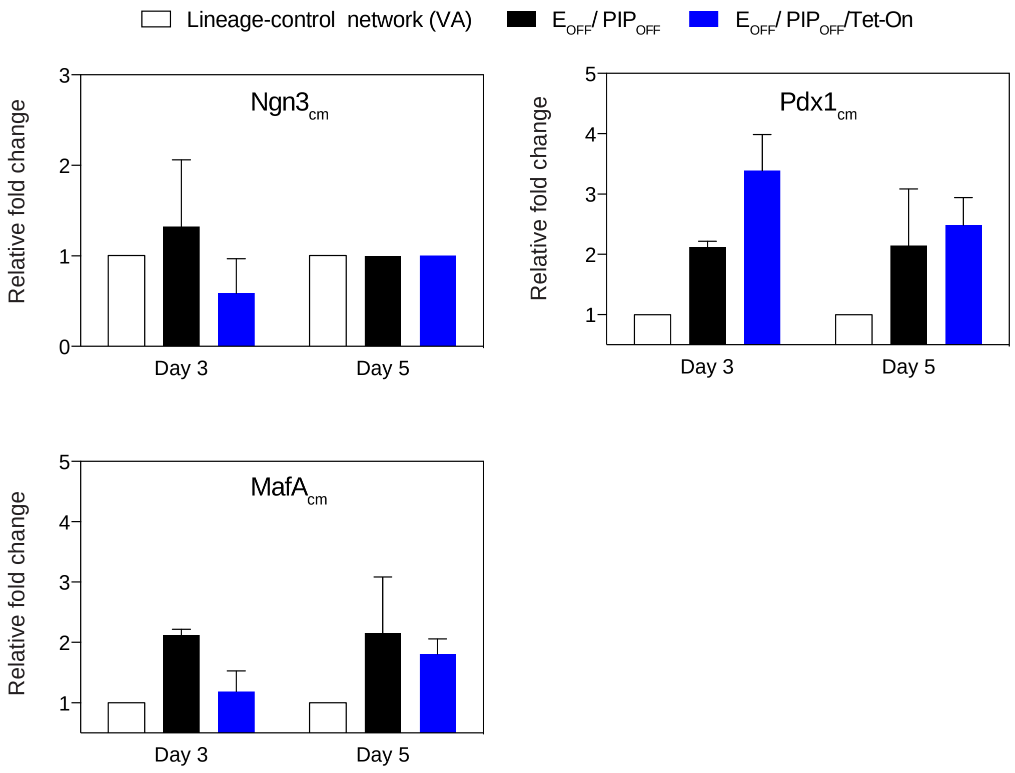

Supplementary Figure 2. Comparative performance of vanillic acid-triggered lineage-control network with antibiotics-controlled gene switches by qRT-PCR-based expression profiling. For lineage control network, $2x10^5$ hMSC-TERT were cotransfected with pCI-MOR9-1 (P_{hCMV}-MOR9-1-pA), pSP1 (P_{CRE}-$VanA_1$-pA) and pSP12 (pA-$Ngn3_{cm}$←P_{3VanO2}→mFT-miR30$Pdx1_{g\text{-}shRNA}$-pA) and pSP17 (P_{CREm}-$Pdx1_{cm}$-2A-$MafA_{cm}$-pA) grown for 3 days in the presence of medium (2μM) and next 2 days in presence of high (400μM) vanillic acid (VA) concentration. For E_{OFF}/PIP_{OFF}, $2x10^5$ hMSC-TERT were cotransfected with pWW35 (P_{SV40}-ET1-pA), pSP36 (P_{ETR2}-$Pdx1_{cm}$-2A-$MafA_{cm}$-pA_{SV40}), pMF156 (P_{SV40}-PIT-pA_{SV40}) and pSP39 (P_{PIR3}-$Ngn3_{cm}$-pA_{SV40}) grown for 3 days in presence of erythromycin (1μM), next 2 days in presence of pristinamycin (1μM). For E_{OFF}/PIP_{OFF}/Tet-ON, $2x10^5$ hMSC-TERT were cotransfected with pWW35 (P_{SV40}-ET1-pA), pSP37 (P_{ETR2}-$Pdx1_{cm}$-pA_{SV40}), pMF156 (P_{SV40}-PIT-pA_{SV40}), pSP39 (P_{PIR3}-$Ngn3_{cm}$-pA_{SV40}), pTet-ON (P_{SV40}-rtTA-pA_{SV40}) and pSP4 ($P_{hCMV*\text{-}1}$-$MafA_{cm}$-pA_{SV40}) grown for 3 days in presence of erythromycin (1μM), next 2 days in presence of pristinamycin (1μM) and doxycycline (1μM).

The transcript levels were profiled relative to the lineage control network and normalised to glyceraldehyde 3-phosphate dehydrogenase (GAPDH). Data are the means ± SD of triplicate experiments (n=9).

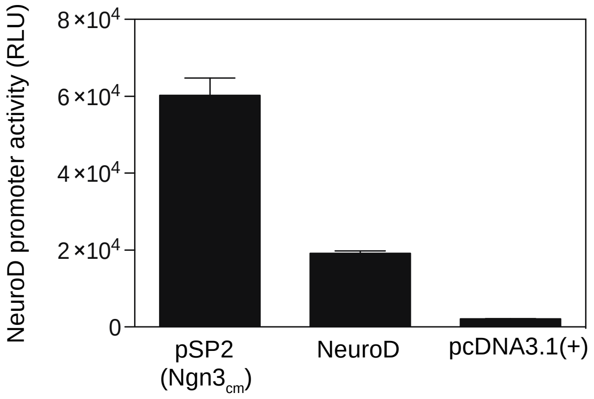

Supplementary Figure 3. Activation of the human NeuroD promoter by Ngn3$_{cm}$ and NeuroD. 2x10^5 hMSC-TERT were cotransfected with the firefly luciferase (FLuc) reporter construct pP$_{E1}$-FLuc (P$_{E1}$-FLuc pA) and either the Ngn3$_{cm}$-encoding pSP2 (P$_{hCMV}$-Ngn3$_{cm}$-pA), the NeuroD-containing pCMV-NeuroD (P$_{hCMV}$-NeuroD-pA) or pcDNA3.1(+) as negative control and grown for 48h before intracellular luciferase was quantified. Data are the means ± SD of triplicate experiments (n=9).

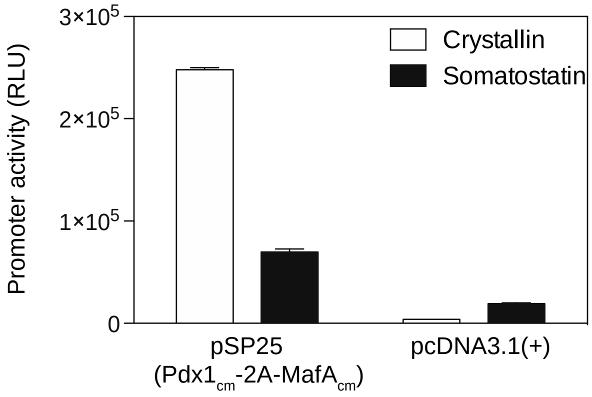

Supplementary Figure 4. Activation of the human somatostatin and crystallin promoters by Pdx1$_{cm}$ and MafA$_{cm}$. 2x10^{5} hMSC-TERT were cotransfected with the constitutive dicistronic Pdx1$_{cm}$ and MafA$_{cm}$ expression vector pSP25 (P$_{hCMV}$-Pdx1$_{cm}$-2A-MafA$_{cm}$-pA) or pcDNA3.1(+) as negative control and either of the somatostatin reporter construct pP$_{SMS900}$-FLuc (P$_{SMS900}$-FLuc- pA) or the crystallin reporter vector pP$_{c\alpha A}$-FLuc (P$_{c\alpha A}$-FLuc-pA) and grown for 48h before intracellular luciferase was quantified. Data are the means ± SD of triplicate experiments (n=9).

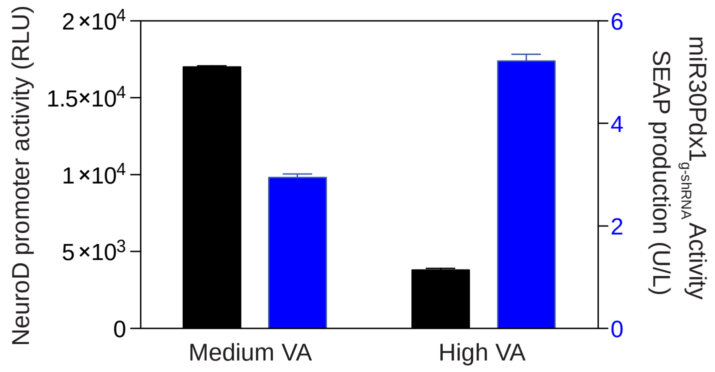

Supplementary Figure 5. Expression levels of Ngn3$_{cm}$ and miR30Pdx1$_{g\text{-}shRNA}$ in the presence of medium and high vanillic acid concentrations. 2x10^{5} hMSC-TERT were cotransfected lineage-control vectors pCI-MOR9-1 (P$_{hCMV}$-MOR9-1-pA), pSP1 (P$_{CRE}$-VanA$_{1}$-pA) and pSP12 (pA-Ngn3$_{cm}$←P$_{3VanO2}$→mFT-miR30Pdx1$_{g\text{-}shRNA}$-pA) as well as either the NeuroD promoter-driven firefly luciferase (FLuc)-encoding reporter (P$_{E1}$-FLuc) or the miR30Pdx1$_{g\text{-}shRNA}$-sensitive SEAP reporter construct pSP14 (P$_{SV40}$ -SEAP-Pdx1$_{UTR}$-pA) and grown for 48h in the presence of medium (2µM) or high (400µM) vanillic acid (VA) concentrations before intracellular luciferase and secreted SEAP was profiled. Data are the means ± SD of triplicate experiments (n=9).

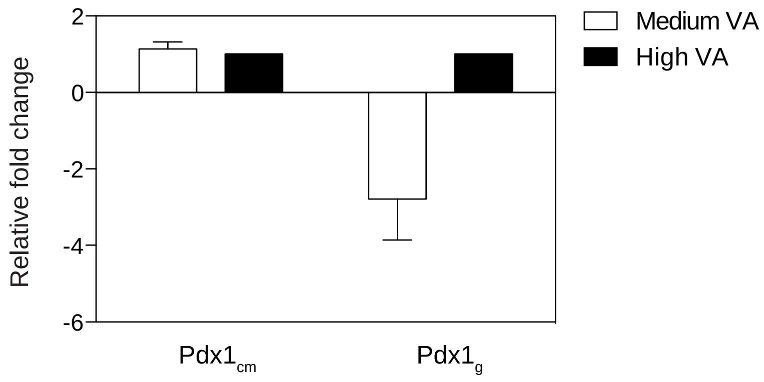

Supplementary Figure 6. Target specificity of miR30Pdx1$_{g\text{-}shRNA}$ in the presence of medium and high vanillic acid concentrations by qRT-PCR-based expression profiling. $2x10^5$ hMSC-TERT were cotransfected lineage-control vectors pCI-MOR9-1 (P_{hCMV}-MOR9-1-pA), pSP1 (P_{CRE}-VanA$_1$-pA) and pSP12 (pA-Ngn3$_{cm}$←P_{3VanO2}→mFT-miR30Pdx1$_{g\text{-}shRNA}$-pA) as well as pSP41 (P_{hCMV}-Pdx1$_{cm}$-pA$_{bGH}$) or pSP42 (P_{hCMV}-Pdx1$_g$-pA$_{bGH}$) and grown for 48h in the presence of medium (2µM) or high (400µM) vanillic acid (VA) concentrations. The transcript levels were profiled relative to the expression with high VA concentration (400µM) and normalised to glyceraldehyde 3-phosphate dehydrogenase (GAPDH). Data are the means ± SD of triplicate experiments (n=9).

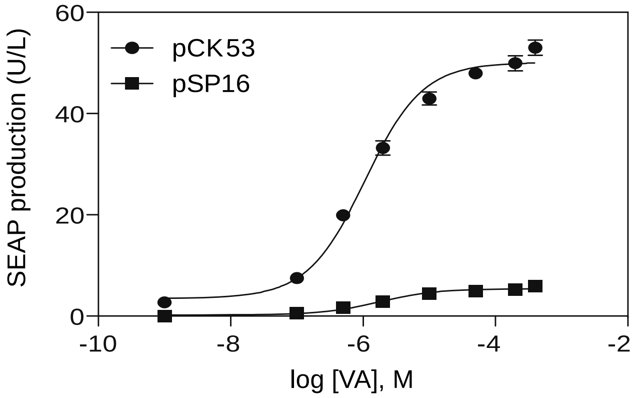

Supplementary Figure 7. Induction kinetics of P_{CRE} and P_{CREm} promoters. 2x10^5 hMSC-TERT were cotransfected with the constitutive MOR9-1 expression vector pCI-MOR9-1 (P_{hCMV}-MOR9-1-pA) and either of the P_{CRE} or P_{CREm}-driven SEAP expression vectors pCK53 (P_{CRE}-SEAP-pA) or pSP16 (P_{CREm}-SEAP-pA) and grown for 48h in the presence of different vanillic acid concentrations before SEAP levels were profiled in the culture supernatant. For comparison, the data using pCK53 (P_{CRE}-SEAP-pA) were replicated from Fig. 1a. Data are the means ± SD of triplicate experiments (n=9).

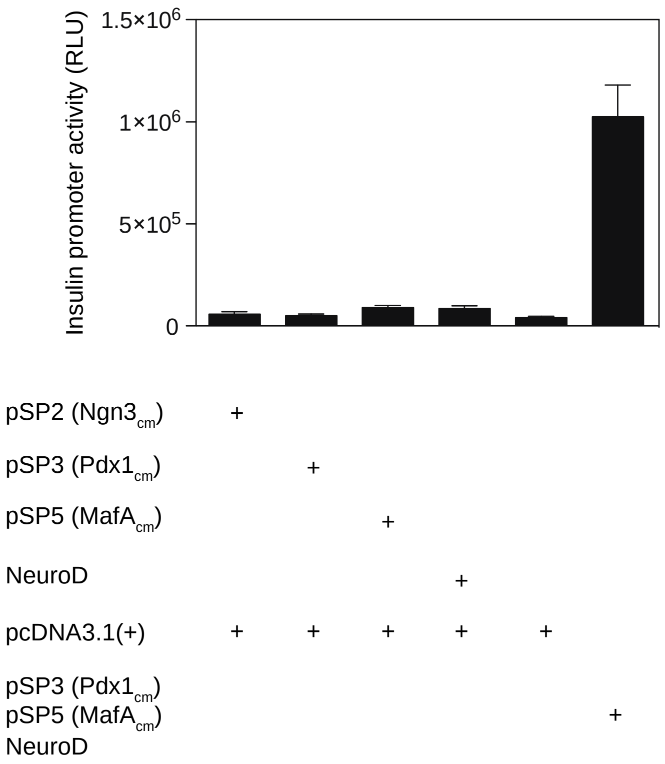

Supplementary Figure 8. Impact of Ngn3, NeuroD, Pdx1 and MafA expression on the activity of the human insulin promoter. $2x10^5$ hMSC-TERT were cotransfected with vectors encoding Ngn3 (pSP2, P_{hCMV}-Ngn3 - pA), Pdx1 (pSP3, P_{hCMV}-Pdx1-pA), MafA (pSP5, P_{hCMV}-MafA-pA), NeuroD (P_{hCMV}-NeuroD - pA), pcDNA3.1(+) and human insulin promoter-driven Gaussia luciferase (GLuc; pSP21 (P_{hINS}-GLuc-pA) and grown for 48h before Gaussia luciferase was profiled in the culture supernatant. Data are the means ± SD of triplicate experiments (n=9).

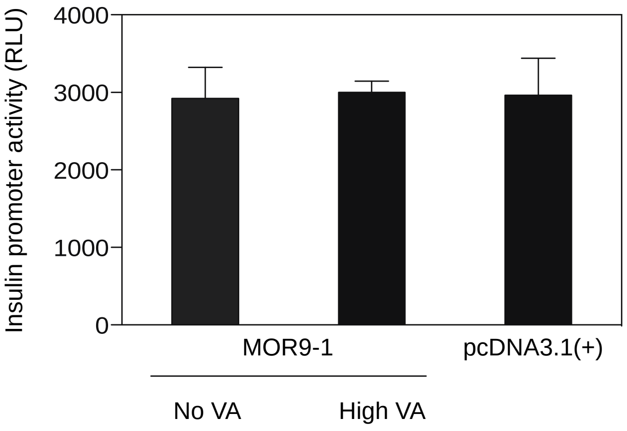

Supplementary Figure 9. Impact of MOR9-1 expression and signaling on the activity of the human insulin promoter. $2x10^5$ hMSC-TERT were cotransfected with pCI-MOR9-1 (P_{hCMV}-MOR9-1-pA) or pcDNA3.1(+) and pSP21 (P_{hINS}-GLuc-pA_{SV40}) and grown for 48h in presence (+; 400µM) and absence of (-) vanillic acid before Gaussia luciferase (GLuc) was profiled in the culture supernatant. Data are the means ± SD of triplicate experiments (n=9).

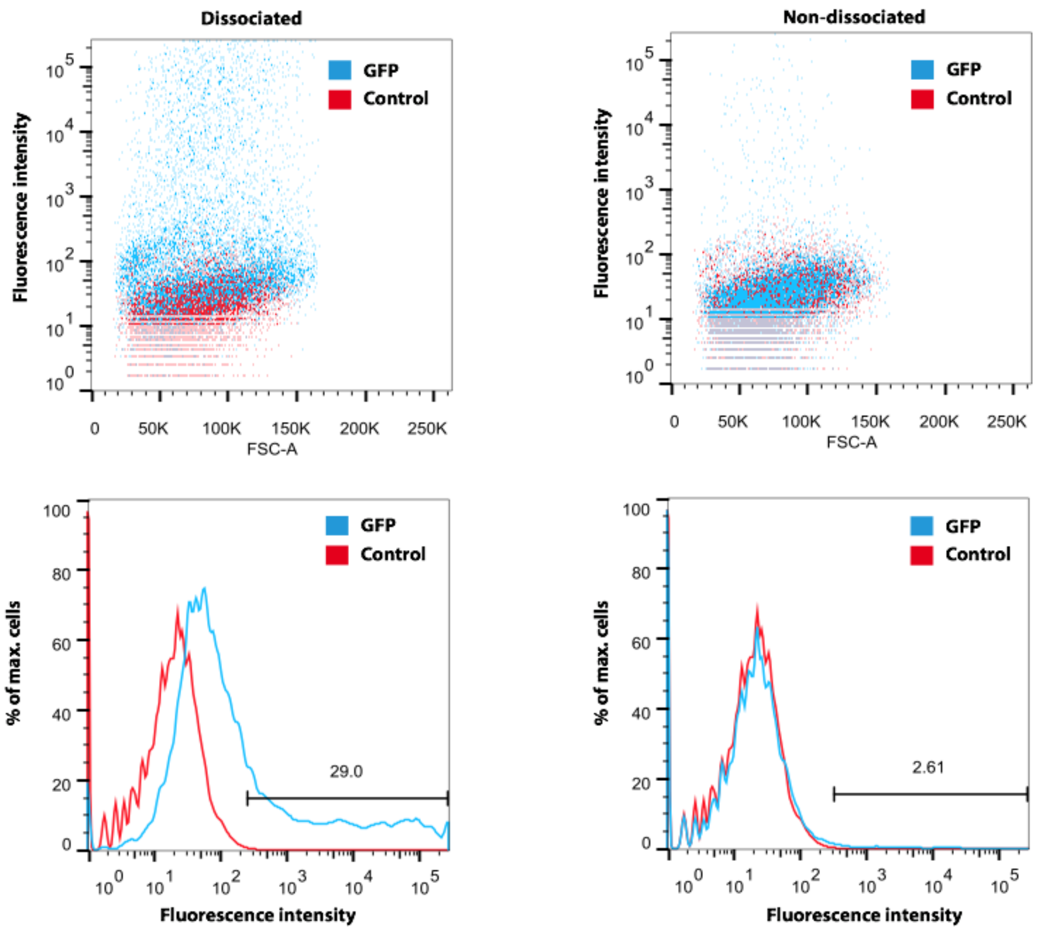

Supplementary Figure 10. FACS-based analysis of the transfection efficiency of pancreatic progenitor cells. Comparative flow-cytometric analysis of dissociated and non-dissociated native and pEGFP-N1-transfected human IPSC-derived pancreatic progenitor cells. Non-transfected pancreatic progenitor cells were used as control.

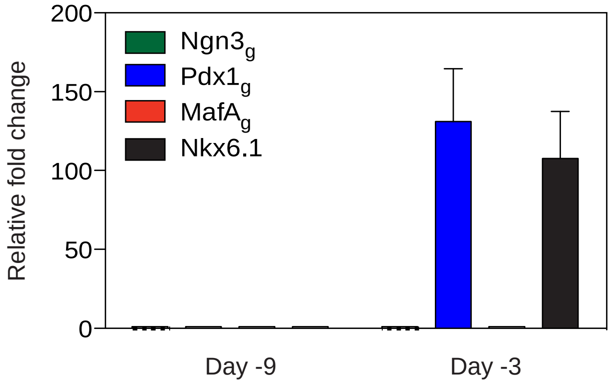

Supplementary Figure 11. qRT-PCR-based expression profiling of Ngn3$_g$, Pdx1$_g$, MafA$_g$ and Nkx6.1 on days -9 and -3. During the differentiation of hIPSCs to pancreatic progenitor cells the expression of the pancreatic transcription factors Ngn3$_g$, Pdx1$_g$ and MafA$_g$ and Nkx6.1 was profiled at days -9 and -3 by qRT-PCR relative to day -13 and normalized to glyceraldehyde 3-phosphate dehydrogenase (GAPDH) expression. Data are the means ± SD of triplicate experiments (n=9).

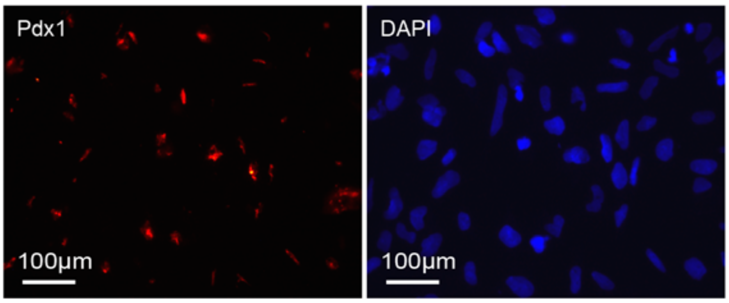

Supplementary Figure 12. Pdx1-specific immunocytochemistry. Pdx1 expression of human IPSC-derived pancreatic progenitor cells was confirmed by immunocytochemistry (day -3). Diamidino-2-phenylindole (DAPI) was used to stain the cell nucleus.

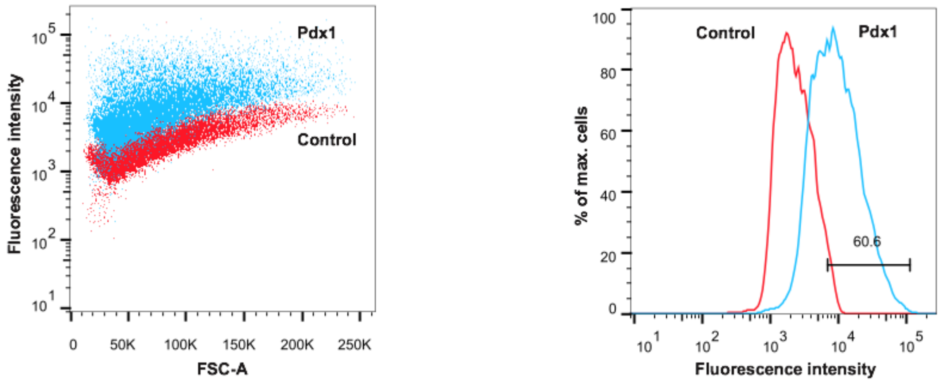

Supplementary Figure 13. FACS-based Pdx1 expression analysis of human IPSC-derived pancreatic progenitor cells. Flow-cytometric analysis of Pdx1 expression in human IPSC-derived pancreatic progenitor cells at day -3. Undifferentiated human IPSCs were used as control.

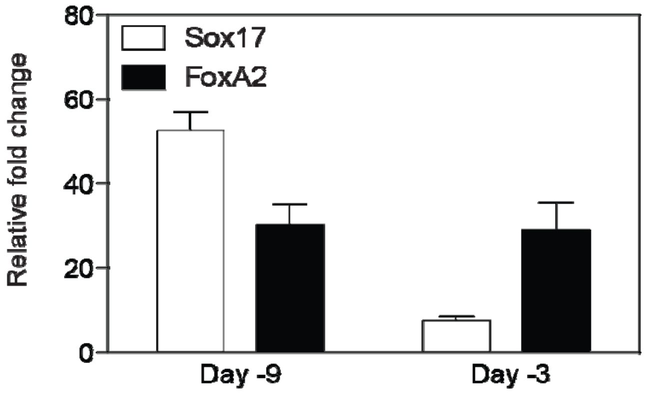

Supplementary Figure 14. qRT-PCR-based expression profiling of Sox17 and FoxA2 on day -9 and day -3.. During the differentiation of hIPSCs to pancreatic progenitor cells the expression of Sox17 and FoxA2 was profiled at day -9 and day -3 by qRT-PCR relative to day -13 (hIPSC) and normalized to glyceraldehyde 3-phosphate dehydrogenase (GAPDH) expression. Data are the means ± SD of triplicate experiments (n=9).

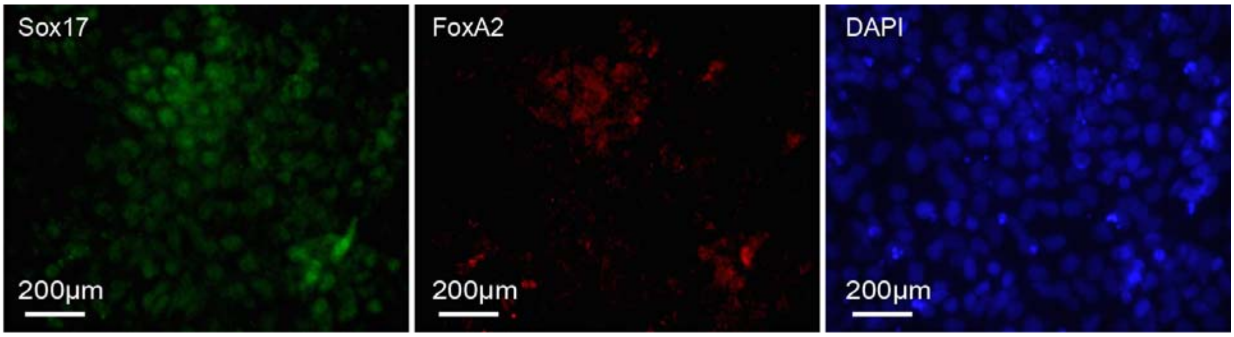

Supplementary Figure 15. Sox17 and FoxA2-specific immunocytochemistry. During the differentiation of human IPSCs to pancreatic progenitor cells the expression of Sox17 and FoxA2 was profiled at day -9. Diamidino-2-phenylindole (DAPI) was used to stain the cell nucleus.

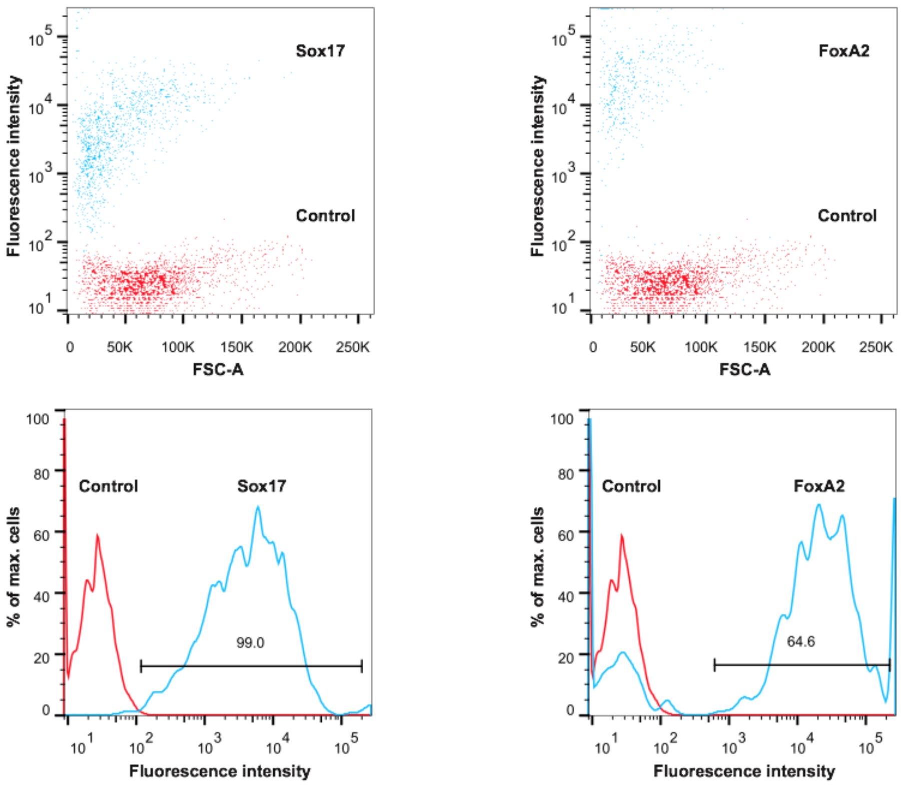

Supplementary Figure 16. FACS-based Sox17 and FoxA2 expression analysis. During the differentiation of human IPSCs to pancreatic progenitor cells the expression of Sox17 and FoxA2 was profiled by FACS analysis at day -9. Undifferentiated human IPSCs were used as control.

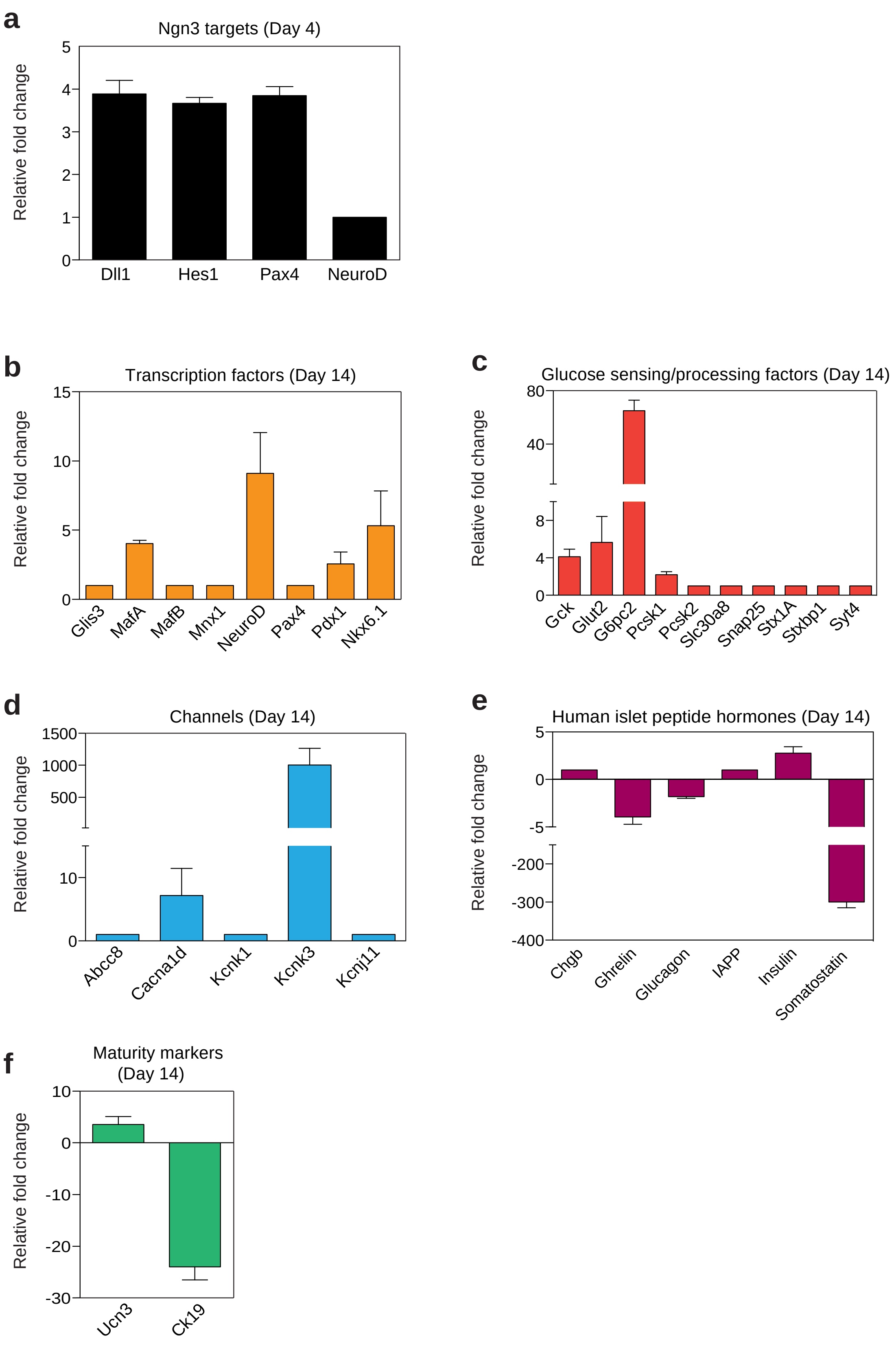
a
Ngn3 targets (Day 4)
Relative fold change
0
1
2
3
4
5
Dll1
Hes1
Pax4
NeuroD
b
Transcription factors (Day 14)
Relative fold change
0
5
10
15
Glis3
MafA
MafB
Mnx1
NeuroD
Pax4
Pdx1
Nkx6.1
c
Glucose sensing/processing factors (Day 14)
Relative fold change
0
4
8
40
80
Gck
Glut2
G6pc2
Pcsk1
Pcsk2
Slc30a8
Snap25
Stx1A
Stxbp1
Syt4
d
Channels (Day 14)
Relative fold change
0
10
500
1000
1500
Abcc8
Cacna1d
Kcnk1
Kcnk3
Kcnj11
e
Human islet peptide hormones (Day 14)
Relative fold change
5
0
-5
-200
-300
-400
Chgb
Ghrelin
Glucagon
IAPP
Insulin
Somatostatin
f
Maturity markers
(Day 14)
Relative fold change
10
0
-10
-20
-30
Ucn3
Ck19

Supplementary Figure 17. Synthetic lineage-control network programming expression of pancreatic transcription factors. a) RT-PCR-based expression profiling of the Ngn3 target genes Dll1, Hes1, Pax4 and NeuroD 4 days after the kick-off of the lineage control network. The transcript levels were profiled at day 4 relative to randomly differentiating cells and normalised to glyceraldehyde 3-phosphate dehydrogenase (GAPDH). Data are the means ± SD of triplicate experiments (n=9). (b) RT-PCR based expression profiling of key β-cell-specific transcription factors Glis3, MafA/B, Mnx1, NeuroD, Pax4, Pdx1 and Nkx6.1 (c) Glucose and insulin processing factors Gck, Glut2, G6pc2, Pcsk1, Pcsk2, Slc30a8, Snap25, Stx1A, Stxbp1, Syt4 (d) RT-PCR based expression profiling of channels essential for the secretion of insulin Abcc8, Cacna1D, Kcnk1/3 and Kcnj11 (e) Human islet peptide hormones Chgb, Ghrelin, Glucagon, Iapp, Insulin and Somatostatin (f) Mature β-cell marker Ucn3 and immature β-cell marker Ck19. The transcript levels were profiled at day 14 relative to randomly differentiating cells and normalised to glyceraldehyde 3-phosphate dehydrogenase (GAPDH). Data are the means ± SD of triplicate experiments (n=9). Abbreviations: Abcc8, ATP-binding cassette transporter sub-family C member 8; Cacna1D, voltage-dependent, L type, alpha 1D subunit; Chgb, Chromogranin B; Ck19, Cytokeratin 19; Dll1, Delta-like1; Gcgr, Glucagon receptor; Gck, Glucokinase; Glut2, Glucose transporter 2; G6pc2, Glucose-6-phosphatase 2; Hes1, Hairy and enhancer of split-1; Iapp, Islet amyloid polypeptide; Kcnk1/3, Potassium channel, subfamily K, member 1/3; Kcnj11, Potassium inwardly-rectifying channel, subfamily J, member 11; MafA/B, V-maf musculoaponeurotic fibrosarcoma oncogene homologue A/B; Mnx1, Motor neuron and pancreas homeobox 1; NeuroD1, Neurogenic differentiation factor 1; Nkx6.1, NK6 homeobox 1; Pcsk1/2, Proprotein convertase 1/2; Pax4, Paired box gene 4; Pdx1, Pancreatic and duodenal homeobox 1; Slc30a8, Solute carrier family 30, member 8; Snap25, Synaptosomal-associated protein; Stx1A, Syntaxin 1A; Stxbp1, Syntaxin binding protein 1; Syt4 Synaptotagmin-4; Ucn3, Urocortin 3.

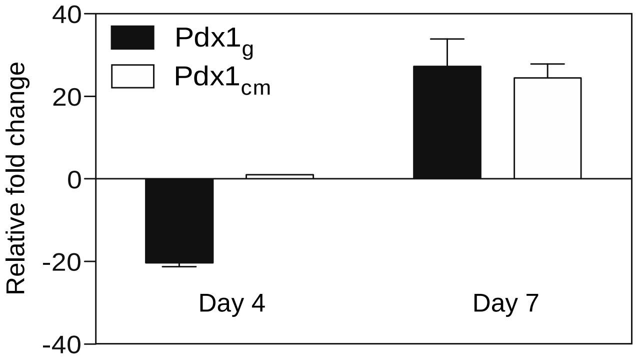

Supplementary Figure 18. Recovery of Pdx1 levels after switching from miR30Pdx1$_{g\text{-}shRNA}$ to Pdx1$_{cm}$ expression. Pancreatic progenitor cells cotransfected with the lineage-control network vectors pCI-MOR9-1 (P$_{hCMV}$-MOR9-1-pA), pSP1 (P$_{CRE}$-VanA$_1$-pA), pSP12 (pA-Ngn3$_{cm}$←P$_{3VanO2}$→mFT-miR30Pdx1$_{g\text{-}shRNA}$-pA) and pSP17 (P$_{CREm}$-Pdx1$_{cm}$-2A-MafA$_{cm}$-pA) were profiled by qRT-PCR for expression of genomic (Pdx1$_g$) and codon-modified (Pdx-1$_{cm}$) Pdx1 on days 4 and 7 relative to randomly differentiating cells and normalized to glyceraldehyde 3-phosphate dehydrogenase (GAPDH) expression. Data are the means ± SD of triplicate experiments (n=9).

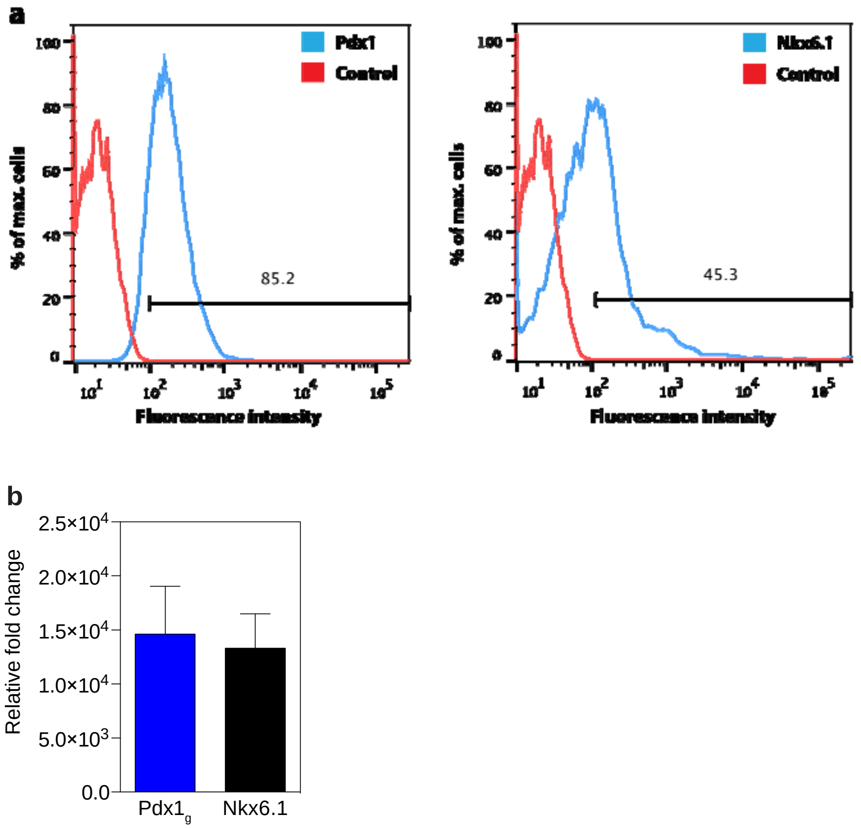

Supplementary Figure 19. Characterization of Pdx1 and Nkx6.1 expression in hIPSC-derived pancreatic progenitor cells differentiated using the growth-factor/chemical-based differentiation technique (Day 0). (a) Flow-cytometric analysis of pancreatic progenitor markers $Pdx1_g$ and Nkx6.1. Undifferentiated hIPSCs were used as negative control. (b) Expression profiling of the pancreatic transcription factors $Pdx1_g$ and Nkx6.1 via qRT-PCR. The transcript levels were profiled relative to the undifferentiated hIPSCs and normalized to GAPDH. Data are the means ± SD of triplicate experiments (n=9).

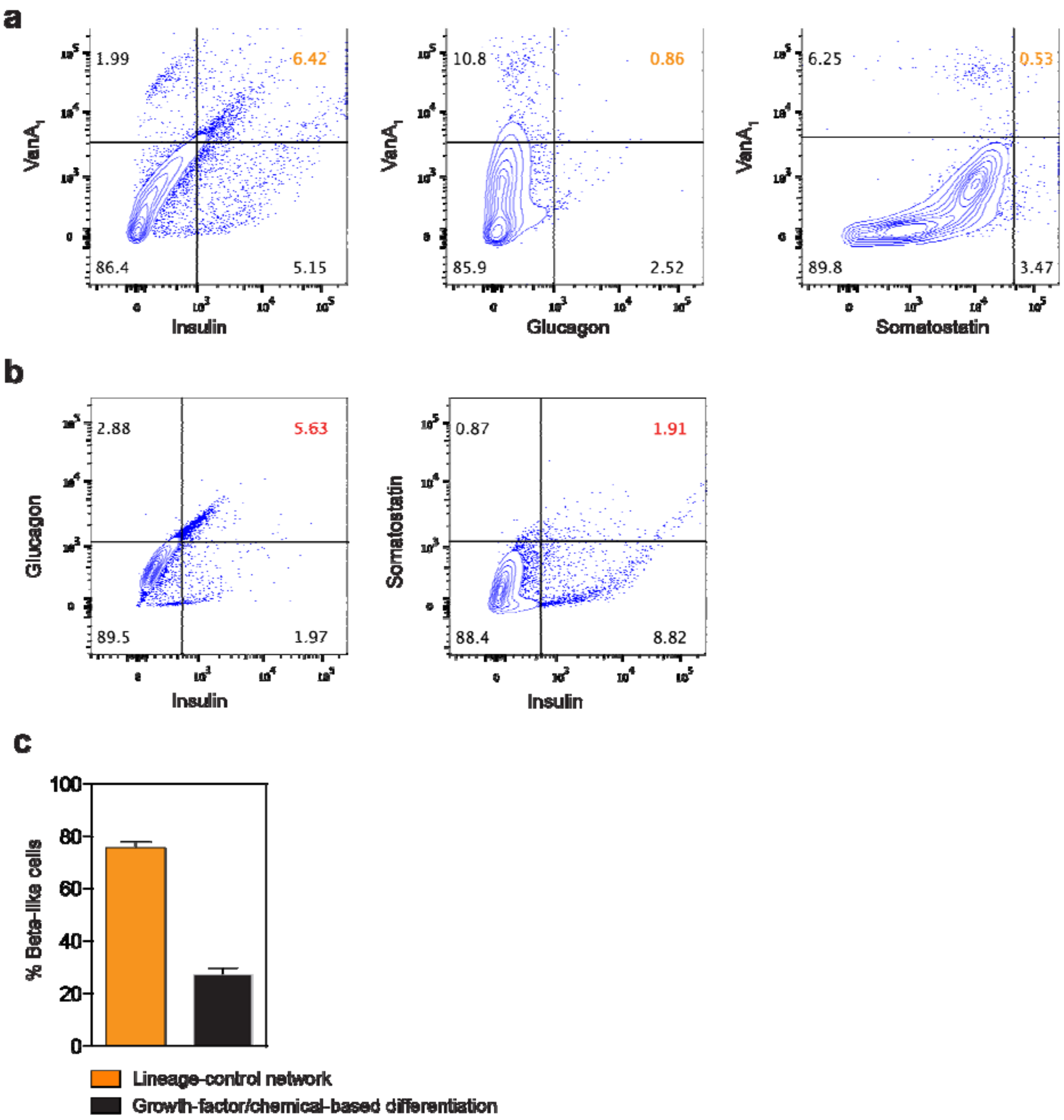

Supplementary Figure 20. Flow-cytometric analysis of beta-like cells differentiated by the synthetic lineage-control network or the growth-factor/chemical-based differentiation technique. (a) Representative panels of flow-cytometric analysis of lineage-controlled beta-like cells co-stained for $VanA_1$ and either insulin (C-peptide), glucagon or somatostatin (orange number indicates percent of co-stained cells). (b) Representative panels of flow-cytometric analysis of growth-factor/chemical-controlled beta-like cells co-stained for insulin (C-peptide) and either glucagon or somatostatin (red number indicates percent of co-stained cells). The

cells staining positive for $VanA_1$ are transgenic for the lineage-control network. (c) Quantitative analysis of the number of beta-like cells produced by the lineage-control network or the growth-factor/chemical-based differentiation technique using the FACS profiles provided in Supplementary Fig. 20a and Supplementary Fig. 20b. For comparison, the data using the lineage-control network were replicated from Figure 5a. Data are the means ± SD (n=3).

Supplementary Table 1. Plasmids and oligonucleotides used and designed in this study

Plasmid	Description and Cloning Strategy	Reference or Source
pcDNA3.1(+)	Constitutive P_{hCMV}-driven mammalian expression vector (P_{hCMV}-MCS-pA_{bGH}).	Invitrogen
$pP_{c\alpha A}$-FLuc	Mammalian crystallin promoter reporter ($P_{c\alpha A}$-FLuc-pA_{SV40}).	(*1*)
pCMV-GLuc2	Constitutive GLuc expression vector (P_{hCMV}-GLuc-pA_{SV40}).	NEB
pCMV-NeuroD	Constitutive NeuroD expression vector. (P_{hCMV}-NeuroD-pA_{SV40}).	GenBank: BC009046; Image ID: 3873419
pd2EYFP-N1	Constitutive D2EYFP expression vector (P_{hCMV}-d2EYFP-pA_{SV40}).	Clontech
pP_{E1}-FLuc	Mammalian NeuroD promoter reporter (P_{E1}-FLuc pA_{SV40})	(*2*)
pEYFP-C1	Constitutive EYFP expression vector (P_{hCMV}-EYFP-pA_{SV40}).	Clontech
pEGFP-N1	Constitutive EGFP expression vector (P_{hCMV}-EGFP-pA_{SV40}).	Clontech
pGL4.23	Expression vector encoding a minimal promoter and FLuc (P_{min}-FLuc-pA_{SV40}).	Promega
pSEAP2-basic	Mammalian SEAP expression vector lacking promoter and enhancer sequences (MCS-SEAP-pA_{SV40}).	Clontech
pSEAP2-control	Constitutive mammalian SEAP expression vector (P_{SV40}-SEAP-pA_{SV40}).	Clontech

pP$_{SMS900}$-FLuc	Mammalian somatostatin promoter reporter (P$_{SMS900}$-FLuc- pA$_{SV40}$).	(*3*)
pTet-On	Constitutive reverse tetracycline-dependent transactivator (P$_{SV40}$-rtTA-pA$_{SV40}$).	Clontech
pTRE-Tight-BI-DsRed-Express	Mammalian expression vector for bidirectional tetracycline-responsive expression of DsRed along with a gene of interest *(*pA$_{SV40}$-DsRed-Express$\leftarrow$P$_{hCMVmin}$-TRE$_{mod}$-P$_{hCMVmin}$$\rightarrow$MCS-pA$_{SV40}$*).*	Clontech
pUC57	pUC19-derived prokaryotic expression vector.	Genescript
pCI-MOR9-1	Constitutive expression vector encoding the mammalian vanillic acid receptor MOR9-1 (P$_{hCMV}$-MOR9-1-pA$_{SV40}$).	(*4*)
pPRIME-TET-GFP-FF3	Lentiviral expression vector encoding tetracycline-responsive expression of EGFP and the miR30-based shRNA targeting FLuc. (5'LTR-P$_{hCMV*-1}$-GFP-miR30$_{FLuc}$-3'LTR).	(*5*)
pTRE-Medium-FT	pTRE-derived mammalian expression vector for tetracycline-responsive expression of the medium blue-to-red fluorescent timer (P$_{hCMV*-1}$-mFT-pA$_{SV40}$).	(*6*)
pWPT-Pdx1	Mammalian expression vector encoding human Pdx1$_{g}$.	(*7*)
pWW35	P$_{SV40}$-driven ET1 expression vector. (P$_{SV40}$-ET1-pA$_{SV40}$).	(*8*)
pWW37	P$_{ETR2}$-driven SEAP expression vector. (P$_{ETR2}$-SEAP-pA$_{SV40}$).	(*8*)

pMF156	P_{SV40}-driven PIT expression vector. (P_{SV40}-PIT-pA_{SV40}).	(*9*)
pMF199	P_{PIR3}-driven SEAP expression vector. (P_{PIR3}-SEAP-pA_{SV40}).	(*9*)
pMC1	Custom-designed pUC57-derived vector containing codon-modified human $Ngn3_{cm}$.	This work
pMC2	Custom-designed pUC57-derived vector containing codon-modified human $Pdx1_{cm}$.	This work
pMC3	Custom-designed pUC57-derived vector containing codon-modified human $MafA_{cm}$.	This work
pCK53	Vector encoding a P_{CRE}-driven SEAP expression unit (P_{CRE}-SEAP-pA_{SV40}).	(*10*)
pMF111	Tetracycline-responsive SEAP expression vector ($P_{hCMV*-1}$-SEAP- pA_{SV40}).	(*11*)
pMG250	Constitutive $VanA_1$ expression vector (P_{SV40}-$VanA_1$-pA_{SV40}).	(*12*)
pMG252	Vector encoding a $VanA_1$-specific vanillic acid-responsive P_{1VanO2}-driven SEAP expression unit (P_{1VanO2}-SEAP-pA_{SV40}).	(*12*)
pMM44	Constitutive mammalian GLuc expression vector. GLuc was PCR-amplified from pCMV-GLuc2 using OMM71 (5'-cggaattcaccggtATGGGAGTCAAAGTTCTGTTTG-3') and OMM72 (5'-gaagatctggccggcctct agaTTAGTCACCACCGGCCCCCTTG-3'), restricted with *Eco*RI/*Xba*I and cloned into the corresponding sites (*Eco*RI/*Xba*I) of pSEAP2-control. (P_{SV40}-GLuc-pA_{SV40}).	This work

pSP1	P_{CRE}-driven $VanA_1$ expression vector. $VanA_1$ was PCR-amplified from pMG250 using OSP1 (5’-acgctcgcgatccaccATGGACATGCCGCGCATAAAGCCGG-3’) and OSP2 (5’- gctgggccggccCTACCCA CCGTACTCGTCAATTCC-3’), restricted with *Nru*I/*Fse*I and cloned into the corresponding sites (*Nru*I/*Fse*I) of pCK53. (P_{CRE}-$VanA_1$-pA_{SV40}).	This work
pSP2	P_{hCMV}-driven $Ngn3_{cm}$ expression vector. $Ngn3_{cm}$ was PCR-amplified from pMC1 using OSP3 (5’-acgcgaattccaccATGACCCCCCAGCCAAGCGGAG-3’) and OSP4 (5’-acgctctagaTTACAGAAAATCGC TAAAAGCCAG- 3’), restricted with *Eco*RI/*Xba*I and cloned into the corresponding sites (*Eco*RI/*Xba*I) of pcDNA 3.1(+). (P_{hCMV}-$Ngn3_{cm}$-pA_{bGH}).	This work
pSP3	P_{hCMV}-driven $Pdx1_{cm}$ expression vector. $Pdx1_{cm}$ was PCR-amplified from pMC2 using OSP5 (5’-acgcgaattccaccATGAACGGGGAGGAACAGTATTATGC-3’) and OSP6 (5’- acgctctagaTTAGCGGGG TTCCTGAGGTCTCCTTG 3’), restricted with *Eco*RI/*Xba*I and cloned into the corresponding sites (*Eco*RI/*Xba*I) of pcDNA 3.1(+). (P_{hCMV}-$Pdx1_{cm}$-pA_{bGH}).	This work
pSP4	$P_{hCMV*-1}$-driven $MafA_{cm}$ expression vector. $MafA_{cm}$ was PCR-amplified from pMC3 using OSP5 (5’-acgcgaattcccaccATGGCTGCTGAACTGGCTATG-3’) and OSP6 (5’-acgcaagcttTTACAGAAAGA AGTCAGCGGT GCC -3’), restricted with *Eco*RI/*Hind*III and cloned into the corresponding sites	This work

	(*Eco*RI/*Hin*dIII) of pMF111. (P$_{hCMV*-1}$-MafA$_{cm}$-pA$_{SV40}$).	
pSP5	P$_{hCMV}$-driven driven MafA$_{cm}$ expression vector. MafA$_{cm}$ was excised from pSP4 using *Eco*RI/*Not*I and cloned into the corresponding sites (*Eco*RI/*Not*I) of pcDNA 3.1(+). (P$_{hCMV}$-MafA$_{cm}$-pA$_{bGH}$).	This work
pSP6	P$_{hCMV*-1}$-driven EGFP and miR30Pdx1$_{g\text{-}shRNA}$ expression vector. The Pdx1$_{g}$-specific hairpin oligonucleotide (5'-TGCTGTTGACAGTGAGCGCGGAGTTCCTATTCAACAAGTATAGTGAAGCCA CAGATGTATACTTGTTGAATAGGAACTCCTTGCCTACTGCCTCGGA-3') was PCR-amplified using OSP7 (5'-gatggctgctcgagAAGGTATATTGCTGTTGACAGTGAGCG-3') and OSP8 (5'-gtctagaggaattcCGAGGCAGTAGGCA-3'), restricted with *Xho*I/*Eco*RI and cloned into the corresponding sites (*Xho*I/*Eco*RI) of pPRIME-TET-GFP-FF3. (P$_{hCMV*-1}$-EGFP-miR30Pdx1$_{g\text{-}shRNA}$-pA$_{SV40}$).	This work
pSP7	P$_{hCMV*-1}$-driven Ngn3$_{cm}$ expression vector. Ngn3$_{cm}$ was excised from pSP2 using *Eco*RI/*Xba*I and cloned into the corresponding sites (*Eco*RI/*Xba*I) of pTRE-Tight-BI-DsRed-Express. (pA$_{SV40}$-Ngn3$_{cm}$←P$_{hCMVmin}$-TRE$_{mod}$-P$_{hCMVmin}$→MCS-pA$_{SV40}$*)*.	This work
pSP8	P$_{1VanO2}$-driven EGFP and miR30Pdx1$_{g\text{-}shRNA}$ expression vector. P$_{1VanO2}$ was PCR-amplified from pMG252 using OSP9 (5'-acgctctagaGTCAATTCGCGAATTGGATCCAATAGCG-3') and OSP10 (5'-gctaaccggtC GCGGAGGCTGGATCGG-3'), restricted with *Xba*I/*Age*I and cloned into the corresponding sites	This work

	(*Xba*I/*Age*I) of pSP6. (P_{1VanO2}-GFP-miR30Pdx$1_{g\text{-}shRNA}$- pA_{SV40}).	
pSP10	P_{1VanO2}-driven mFT and miR30Pdx$1_{g\text{-}shRNA}$ expression vector. mFT was PCR-amplified from pTRE-Medium-FT using OSP13 (5’-gcatgaattcaccggtcgccacc ATGGTGAGCAAGGGCGAGGAGGATAAC-3’) and OSP14 (5’-gcattctaga gcggccgcTTACTTGTACAGCTCGTCCATG-3’), restricted with (*Age*I/*Not*I) and cloned into the corresponding sites (*Age*I/*Not*I) of pSP8. (P_{1VanO2}-mFT-miR30Pdx$1_{g\text{-}shRNA}$-pA_{SV40}).	This work
pSP12	P_{3VanO2}-driven Ngn3_{cm}, mFT and miR30Pdx$1_{g\text{-}shRNA}$ expression vector. P_{1VanO2}-mFT-mir30Pdx$1_{g\text{-}shRNA}$ was PCR-amplified from pSP10 using OSP15 (5’-acgcctcgagGTCAATTCGCGAATTGGATCCAATAGCG-3’) and OSP16 (5’-acgcaagcttCGCGTCGCCGCGTGTTTAAACGCATTAG-3’), restricted with *Psp*XI/*Hin*dIII and cloned into the corresponding sites (*Psp*XI/*Hin*dIII) of pSP7. (pA_{SV40} - Ngn$3_{cm}$$\leftarrow$$P_{3VanO2}$$\rightarrow$mFT-miR30Pdx$1_{g\text{-}shRNA}$-$pA_{SV40}$).	This work
pSP14	SEAP2-control-based expression vector encoding a miR30Pdx$1_{g\text{-}shRNA}$-sensitive SEAP transcription unit linked to Pdx1_{UTR}. SEAP was PCR-amplified from pSEAP2-control using OSP17 (5’-acgcgaattcGCCCACCATGCTGC-3’) and OSP18 (5’- acgctctaga tacttgttgaataggaactccttTCATGTCTGCT CGAAGCGGCCGGCCGCCCCGACTCTTG-3’), restricted with *Eco*RI/*Xba*I and cloned into the corresponding sites (*Eco*RI/*Xba*I) of pSEAP2-control. (P_{SV40} -SEAP-Pdx1_{UTR}-pA_{SV40}).	This work

pSP15	pSEAP2-basic containing CREm. OSP19 (5'-acgcgctagcAGCCTGACGTCCGAGAGCCTGACGTCCGAGAGCCTGACGTCCGAGAGCCTGACGTCCGAGATCTCTCGAGGTCGACAGCGGAGACTCTAGAGGGTATATAgaattcacgc-3') and OSP191 (5'-gcgtgaattcTATATACCCTCTAGAGTCTCCGCTGTCGACCTCGAGAGATCTCGGACGTCAGGCTCTCGGACGTCAGGCTCTCGGACGTCAGGCTCTCGGACGTCAGGCTgctagcgcgt-3') were annealed, restricted with *Nhe*I/*Eco*RI and cloned into the corresponding sites (*Nhe*I/*Eco*RI) of pSEAP2-basic. (CREm-SEAP-pA_{SV40}).	(13)
pSP16	pSEAP2-basic containing a P_{CREm}-driven SEAP expression unit. pGL4.23-derived P_{min} encoded by OSP20 (5'atcgctcgagGTCGACAGCGGAGACTCTAGAGGGTATATAATGGAAGCTCGACTTCCAGCTTGGCAATCCGGTACTGTTGGTAAAgaattcatcg-3') and OSP21 (5'-cgatgaattcTTTACCAACAGTACCGGATTGCCAAGCTGGAAGTCGAGCTTCCATTATATACCCTCTAGAGTCTCCGCTGTCGACctcgagcgat-3'), restricted with *Xho*I/*Eco*RI and cloned into the corresponding sites (*Xho*I/*Eco*RI) of pSP15. (P_{CREm}-SEAP-pA_{SV40}).	(13)
pSP17	Dicistronic P_{CREm}-driven $Pdx1_{cm}$ and $MafA_{cm}$ expression vector. $Pdx1_{cm}$-2A was PCR-amplified from pSP3 using OSP5 (5'-acgcgaattccaccATGAACGGGGAGGAACAGTATTATGC-3') and OSP22 (5'-aggtccagggttggactccacgtctcccgccaacttgagaaggtcaaaattcaacaaGCGGGGTTCCTGAGGTCTCCTTG-3').	This work

	2A-MafA$_{cm}$ was PCR-amplified from pSP5 using OSP23 (5'-ttgttgaattttgaccttctcaagttggcgggagacgtggagtc caaccctggacctATGGCTGCTGAACTGGCTATG-3') and OSP24 (5'-gcatgcgcgctctagattaCAGAAAGAA GTCAGCGGTGCC-3'). Both PCR fragments were annealed and Pdx1$_{cm}$-2A-MafA$_{cm}$ was PCR-amplified using OSP5 and OSP24, restricted with *Eco*RI/*Bss*HII and cloned into the corresponding sites (*Eco*RI/*Bss*HII) pSP16. (P$_{CREm}$-Pdx1$_{cm}$-2A-MafA$_{cm}$-pA$_{SV40}$).	
pSP19	pUC57 containing the human insulin promoter (P$_{hINS}$; -881 to +54).	This work
pSP21	P$_{hINS}$-driven GLuc expression vector. P$_{hINS}$ was excised from pSP19 with *Xho*I/*Eco*RI and cloned into the corresponding sites (*Xho*I/*Eco*RI) of pMM44. (P$_{hINS}$-GLuc-pA$_{SV40}$).	This work
pSP24	P$_{CREm}$-driven EYFP expression vector. P$_{CREm}$ was excised from pSP15 with *Mlu*I/*Eco*RI and EYFP was PCR-amplified from pEYFP-C1using OMM48 (5'- ggaattcactagtgcccggggaaccggtATGGTGAGCAAGG GCGAG-3') and OMM54 (5'-gctctagatctggccggccctaTTACTTGTACAGCTCGTCCATG-3'), restricted with *Eco*RI/*Xba*I and both fragments were cloned into the compatible sites (*Mlu*I/*Xba*I) of pSEAP2-basic. (P$_{CREm}$-EYFP- pA$_{SV40}$).	This work
pSP25	Dicistronic P$_{hCMV}$-driven Pdx1$_{cm}$-2A-MafA$_{cm}$ expression vector. Pdx1$_{cm}$-2A-MafA$_{cm}$ was excised from pSP17 with *Eco*RI/*Xba*I and cloned into the corresponding sites (*Eco*RI/*Xba*I) of pcDNA3.1(+). (P$_{hCMV}$-	This work

	Pdx1$_{cm}$-2A-MafA$_{cm}$ -pA$_{SV40}$).	
pSP26	P$_{hINS}$-driven DsRed-Express expression vector. DsRed-Express was PCR-amplified from pTRE-Tight-BI-DsRed-Express using OSP25 (5'-acgcgaattcgccaccATGGCCTCCTCCGAGGACGTC-3') and OSP26 (5'-acgctctagaCTACAGGAACAGGTGGTGGCG-3'), restricted with (*EcoRI*/*XbaI)* and cloned into the corresponding sites (*EcoR*I/*XbaI*) of pSP21. (P$_{hINS}$-DsRed-Express-pA$_{SV40}$).	This work
pSP36	Dicistronic P$_{ETR2}$-driven Pdx1$_{cm}$-2A-MafA$_{cm}$ expression vector. Pdx1$_{cm}$-2A-MafA$_{cm}$ was excised from pSP25 with *Eco*RI/*Xba*I and cloned into the corresponding sites (*Eco*RI/*Xba*I) of pWW37. (P$_{ETR2}$-Pdx1$_{cm}$-2A-MafA$_{cm}$ -pA$_{SV40}$).	This work
pSP37	P$_{ETR2}$-driven Pdx1$_{cm}$ expression vector. Pdx1$_{cm}$ was PCR-amplified from pSP36 using OSP80 (5'-acgcgaattcGCCACCATGAACGGGGAGGAACAGTATTATG-3') and OSP81 (5'-acgctctagaTCA GCGGGGTTCCTGAGGTCTCCTTG-3'), restricted with *Eco*RI/*Xba*I and cloned into the corresponding sites (*Eco*RI/*Xba*I) of pWW37. (P$_{ETR2}$-Pdx1$_{cm}$-pA$_{SV40}$).	This work
pSP39	P$_{PIR3}$-driven Ngn3$_{cm}$ expression vector. Ngn3$_{cm}$ was excised from pSP12 with *Eco*RI/*Xba*I and cloned into the corresponding sites (*Eco*RI/*Xba*I) of pMF199. (P$_{PIR3}$-Ngn3$_{cm}$-pA$_{SV40}$).	This work
pSP41	P$_{hCMV}$-driven Pdx1$_{cm}$ expression vector. Pdx1$_{cm}$ was excised from pSP37 with *Eco*RI/*Xba*I and cloned	This work

	into the corresponding sites (*Eco*RI/*Xba*I) of pcDNA3.1(+) (P_{hCMV}-Pdx1$_{cm}$-pA$_{bGH}$).	
pSP42	P_{hCMV}-driven Pdx1$_{g}$ expression vector. Pdx1$_{g}$ was PCR-amplified from pWPT-Pdx1 using OSP82 (5'-acgcgaattcGCCACCATGAACGGGGAGGAACAGTATTATG-3') and OSP83 (5'-acgctctagaTCAGCGGGGTTCCTGAGGTCTCCTTG-3'), restricted with *Eco*RI/*Xba*I and cloned into the corresponding sites (*Eco*RI/*Xba*I) of pWW37 (P_{hCMV}-Pdx1$_{g}$-pA$_{bGH}$).	This work

Oligonucleotides: restriction endonuclease-specific sites are underlined in oligonucleotide sequences. Annealing base pairs contained in oligonucleotide sequences are shown in capital letters.

Abbreviations: **DsRed-Express**, rapidly maturing variant of the *Discosoma* sp. red fluorescent protein; **EGFP**, enhanced green fluorescent protein; **ET1**, macrolide-dependent transactivator (E-VP16); **ETR**, ET1-specific operator sequence; **EYFP**, enhanced yellow fluorescent protein; **FLuc**, firefly *Photinus pyralis* luciferase; **GFP**, green fluorescent protein; **GLuc**, *Gaussia princeps* secreted luciferase; **CRE$_{m}$**, modified cAMP response element; **MafA$_{cm}$**, codon-modified v-maf musculoaponeurotic fibrosarcoma oncogene homolog A sequence encoding native MafA by a sequence that is distinct from chomosomally encoded MafA; **MCS**, multiple cloning site; **mFT**, mCherry-derived medium blue-to-red fluorescent timer; **miR30$_{Fluc}$**, microRNA30 containing a firefly luciferase-specific small hairpin RNA; **miR30Pdx1$_{g\text{-}shRNA}$,** microRNA30-derived Pdx1$_{g}$-specific small hairpin RNA; **MOR9-1**, mammalian olfactory receptor 9, a N-terminally rhodopsin-tagged GPCR responsive to vanillic acid; **NeuroD**, Neurogenic differentiation 1; **Ngn3$_{cm}$**, codon-modified neurogenin 3 sequence encoding native Ngn3 by a

sequence that is distinct from chromosomally encoded Ngn3; **pA_{bGH}**, polyadenylation signal of the bovine growth hormone; **pA_{SV40}**, polyadenylation signal of the simian virus 40; **$P_{c\alpha A}$**, mammalian αA-crystallin promoter; **P_{CRE}**, synthetic mammalian promoter containing a cAMP-response element (CRE-$P_{hCMVmin}$); **P_{CREm}**, synthetic mammalian promoter containing a modified cAMP-response element (CRE_m-P_{min}); **$Pdx1_{cm}$**, codon-modified sequence of the pancreatic and duodenal homeobox 1 encoding native Pdx1 protein by a distinct sequence differing from $Pdx1_g$.; **$Pdx1_g$,** genomic sequence of the pancreatic and duodenal homeobox 1 encoding native Pdx1 by the wild-type sequence that differs from $Pdx1_{cm}$; **P_{E1}**, mammalian NeuroD E-box promoter; **P_{ETR2}**, ET1-specific macrolide-responsive promoter (ETR-$P_{hCMVmin}$), **P_{hCMV}**, human cytomegalovirus immediate early promoter; **$P_{hCMVmin}$**, minimal version of human cytomegalovirus immediate early promoter; **$P_{hCMV*-1}$**, tetracycline-responsive promoter ($tetO_7$-$P_{hCMVmin}$); **$P_{hCMVtight}$**, modified variant of $P_{hCMVmin}$; **P_{hINS}**, human insulin promoter (-881 to +54); **P_{min}**, pGL4.23-derived minimal promoter; **P_{PIR3}**, PIT-specific streptogramin-responsive promoter (PIT-$P_{hCMVmin}$); **P_{SMS900}**, mammalian somatostatin promoter; **$Pdx1_{UTR}$**, Pdx1-derived untranslated region; **PIT**, streptogramin-dependent transactivator (Pip-VP16)**; PIR**, PIT-specific operator sequence; **P_{1VanO2}**; vanillic acid-responsive promoter ($VanO_2$-$P_{hCMVmin}$); **P_{3VanO2},** vanillic acid responsive promoter ($P_{hCMVtight}$-$VanO_2$-$P_{hCMVmin}$); **rtTA**, reverse tetracycline-dependent transactivator (rTetR-VP16); **TetR**, *Escherichia coli Tn*10-derived tetracycline repressor; **TRE_{mod},** Tet response element; **SEAP**, human placental secreted alkaline phosphatase; **$tetO_7$**, heptameric TetR-specific operator module; **VanA1**; vanillic acid-dependent transactivator (VanR-VP16); **$VanO_n$**, synthetic operator module containing n VanR-specific operators; **VanR**, repressor of the *Caulobacter crescentus* VanAB gene cluster; **VP16**, *Herpes simplex* virus protein 16; **2A,** foot and mouth disease virus-derived 2A sequence. **5'/3' LTR**, human immunodeficiency virus long terminal repeat.

Supplementary Table 2. Taqman primers and qRT-PCR probes

Gene	Gene ID	Assay ID	Dye
Abcc8	6833	Hs01093761_m1	FAM
Acox2	8309	Hs00185873_m1	FAM
Cacna1D	776	Hs01073321_m1	FAM
Chgb	1114	Hs01084631_m1	FAM
Ck19	3880	Hs00761767_s1	FAM
Dll1	28514	Hs00194509_m1	FAM
Dpp4	1803	Hs00897391_m1	FAM
FoxA1	3169	Hs04187555_m1	FAM
FoxA2	3170	Hs00232764_m1	FAM
Fzd2	2535	Hs00361432_s1	FAM
Gapdh	2597	Hs02758991_g1	FAM
Gcgr	2642	Hs01026189_g1	FAM
Gck	2645	Hs01564555_m1	FAM

Ghrelin	51738	Hs01074053_m1	FAM
Glis3	169792	Hs00541450_m1	FAM
Glucagon	2641	Hs01031536_m1	FAM
Glut2	6514	Hs01096904_m1	FAM
G6PC2	57818	Hs01549773_m1	FAM
Hes1	3280	Hs00172878_m1	FAM
Iapp	3375	Hs00169095_m1	FAM
Insulin	3630	Hs02741908_m1	FAM
Irx2	153572	Hs01383002_m1	FAM
Kcnj11	3767	Hs00265026_s1	FAM
Kcnk1	3775	Hs00158428_m1	FAM
Kcnk3	3777	Hs00605529_m1	FAM
MafA	389692	Hs01651425_s1	FAM
MafB	9935	Hs00534343_s1	FAM
Mmp2	4313	Hs01548727_m1	FAM
Mnx1	3110	Hs00907365_m1	FAM

NeuroD1	4760	Hs01922995_s1	FAM
Ngn3	50674	Hs01875204_s1	FAM
Nkx6.1	4825	Hs00232355_m1	FAM
Onecut2	9480	Hs00191477_m1	FAM
Pax4	5078	Hs00173014_m1	FAM
Pcsk1	5122	Hs01026107_m1	FAM
Pcsk2	5126	Hs01037347_m1	FAM
Sftpd	6441	Hs01108490_m1	FAM
Pdx1	3651	Hs00236830_m1	FAM
Slc30a8	169026	Hs00545183_m1	FAM
Snap25	6616	Hs0093962_m1	FAM
Stxbp1	6812	Hs01119036_m1	FAM
Stx1A	6804	Hs00270282_m1	FAM
Somatostatin	6750	Hs00356144_m1	FAM
Sox17	64321	Hs00751752_s1	FAM
Syt4	6860	Hs01086433_m1	FAM

Ucn3	114131	Hs00846499_s1	FAM

Supplementary Table 3. Custom-designed qRT-PCR primers.

$Ngn3_{cm}$ Fw	5'-AGACTGAACGGTCCTTCCCT-3'
$Ngn3_{cm}$ Rv	5'-TGCATCCTATTTCTTTCCCG-3'
$Pdx1_{cm}$-2A-$MafA_{cm}$ Fw	5'-AAGTTGGCGGGAGACGTGGA-3'
$Pdx1_{cm}$-2A-$MafA_{cm}$ Rv	5'-AGATTCAGGGCTTCAGGGTT-3'
$Pdx1_{cm}$ Fw	5'-ACCCGATATCAGTCCCTACG-3'
$Pdx1_{cm}$ Rv	5'-CTCTGCTCCTTCTGGGAAAG-3'
$MafA_{cm}$ Fw	5'-AGGAGGAGGAGCAGACGATA-3'
$MafA_{cm}$ Rv	5'-GGTGATGATGGTGGTGATGT-3'
$Pdx1_{g}$ Fw	5'-CTGCTAGAGCTGGAGAAGGAG-3'
$Pdx1_{g}$ Rv	5'-CAAGTTCAACATGACAGCCA-3

Supplementary References

1. Yoshida T, Yasuda K. Characterization of the chicken L-Maf, MafB and c-Maf in crystallin gene regulation and lens differentiation. *Genes to cells : devoted to molecular & cellular mechanisms* **7**, 693-706 (2002).
2. Huang HP, Liu M, El-Hodiri HM, Chu K, Jamrich M, Tsai MJ. Regulation of the pancreatic islet-specific gene BETA2 (neuroD) by neurogenin 3. *Molecular and cellular biology* **20**, 3292-3307 (2000).
3. Schwartz PT, Vallejo M. Differential regulation of basal and cyclic adenosine 3',5'-monophosphate-induced somatostatin gene transcription in neural cells by DNA control elements that bind homeodomain proteins. *Molecular endocrinology* **12**, 1280-1293 (1998).
4. Saito H, Chi Q, Zhuang H, Matsunami H, Mainland JD. Odor coding by a Mammalian receptor repertoire. *Science signaling* **2**, ra9 (2009).
5. Stegmeier F, Hu G, Rickles RJ, Hannon GJ, Elledge SJ. A lentiviral microRNA-based system for single-copy polymerase II-regulated RNA interference in mammalian cells. *Proceedings of the National Academy of Sciences of the United States of America* **102**, 13212-13217 (2005).
6. Subach FV*, et al.* Monomeric fluorescent timers that change color from blue to red report on cellular trafficking. *Nature chemical biology* **5**, 118-126 (2009).
7. Ritz-Laser B*, et al.* Ectopic expression of the beta-cell specific transcription factor Pdx1 inhibits glucagon gene transcription. *Diabetologia* **46**, 810-821 (2003).
8. Weber W*, et al.* Macrolide-based transgene control in mammalian cells and mice. *Nature biotechnology* **20**, 901-907 (2002).
9. Fussenegger M*, et al.* Streptogramin-based gene regulation systems for mammalian cells. *Nature biotechnology* **18**, 1203-1208 (2000).
10. Kemmer C, Fluri DA, Witschi U, Passeraub A, Gutzwiller A, Fussenegger M. A designer network coordinating bovine artificial insemination by ovulation-triggered release of implanted sperms. *Journal of controlled release : official journal of the Controlled Release Society* **150**, 23-29 (2011).
11. Fussenegger M, Mazur X, Bailey JE. A novel cytostatic process enhances the productivity of Chinese hamster ovary cells. *Biotechnology and bioengineering* **55**, 927-939 (1997).
12. Gitzinger M, Kemmer C, Fluri DA, El-Baba MD, Weber W, Fussenegger M. The food additive vanillic acid controls transgene expression in mammalian cells and mice. *Nucleic acids research* **40**, e37 (2012).
13. Saxena P, Charpin-El Hamri G, Folcher M, Zulewski H, Fussenegger M. Synthetic gene network restoring endogenous pituitary-thyroid feedback control in experimental Graves' disease. *Proceedings of the National Academy of Sciences of the United States of America* **113**, 1244-1249 (2016).
